# Antiprotozoal Activity of Highly Substituted Pyrazole and Pyrimidine Derivatives

**DOI:** 10.1002/cmdc.202500154

**Published:** 2025-06-29

**Authors:** Matteo Lusardi, Nicoletta Basilico, Erika Iervasi, Chiara Brullo, Silvia Parapini, Marco Ponassi, Camillo Rosano, Andrea Spallarossa

**Affiliations:** ^1^ Department of Pharmacy Università degli Studi di Genova viale Benedetto XV, 3 16132 Genova Italy; ^2^ Molecular Modeling and Drug Discovery Laboratory Istituto Italiano di Tecnologia Via Morego, 30 16163 Genova Italy; ^3^ Dipartimento di Scienze Biomediche Chirurgiche e Odontoiatriche Università degli Studi di Milano Via della Commenda, 10 20133 Milan Italy; ^4^ Proteomics and Mass Spectrometry Unit IRCCS Ospedale Policlinico San Martino L.go R. Benzi, 10 16132 Genova Italy; ^5^ Dipartimento di Scienze Biomediche per la Salute Università degli Studi di Milano Via Pascal, 36 20133 Milan Italy

**Keywords:** antileishmanial agents, antimalarial agents, pyrazole compounds, pyrimidine derivatives

## Abstract

To further extend the structure‐activity relationships of previously reported antimalarial anilino‐pyrazoles **VI**, trisubstituted pyrazoles **13–15,** and pyrimidines **16** and **17** are designed and synthesized. The novel derivatives are prepared thorough a divergent, chemo‐selective approach starting from N,S‐acetal intermediates. Compounds **13–17** are tested for their antimalarial and antileishmanial activity and their cytotoxicity is evaluated against human fibroblast. Pyrazoles **14 d,e** and pyrimidine **17e** are identified as novel and effective antiplasmodial agents being able to inhibit, at micromolar concentrations, chloroquine(CQ)‐sensitive and CQ‐resistant *Plasmodium falciparum* strains, as well as *Leishmania infatum* and *Leishmania tropica* protozoa. Additionally, favorable pharmacokinetics and toxicity profiles are predicted for the compounds.

## Graphical abstract

Based on previously identified antimalarial agents, a series of highly substituted pyrazole and pyrimidines derivatives has been synthesized and tested for antiplasmodial and antileishmanial activity. Selected derivatives show promising antiplasmodial effect with a predicted favorable pharmacokinetic profile.

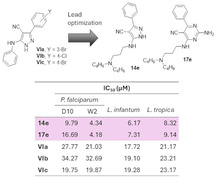

## 1 Introduction

Vector‐borne protozoan infections are responsible for a wide variety of diseases, mainly affecting tropical and subtropical areas but increasingly diagnosed in nonendemic countries.^[^ ^1^, ^2^ ^]^ Along with African sleeping sickness, Chagas’ disease, amebic dysentery, and toxoplasmosis, malaria and leishmaniasis significantly contribute to the burden of protozoal diseases worldwide.^[^ ^3^ ^]^ Furthermore, it has been hypothesized that a possible correlation between leishmaniasis and cancers maybe through alterations in DNA methylation.^[^ ^4^ ^]^

Malaria is a life‐threatening disease caused by protozoan parasites of the *Plasmodium* genus. Commonly, six species infect humans (namely, *Plasmodium falciparum*, *Plasmodium vivax*, *Plasmodium malariae*, *Plasmodium ovale wallikeri*, *Plasmodium ovale curtisi*, and *Plasmodium knowlesi*), being *Plasmodium falciparum* and *Plasmodium vivax* the most clinically significant.^[^ ^5^ ^]^ In particular, *P. falciparum* accounts for 97% of global malaria cases and represents the most likely species to cause severe illness.^[^ ^6^ ^]^ *P. vivax* is endemic in South America and South‐East Asia and constitutes the most geographically widespread species.^[^ ^7^ ^]^ In 2022 249 millions of malaria cases were estimated worldwide with an increase of 5 million cases compared with 2021.^[^ ^8^ ^]^ The main countries contributing to this increase of cases were Pakistan (+2.1 million), Ethiopia (+1.3 million), Nigeria (+1.3 million), Uganda (+597 000) and Papua New Guinea (+423 000). 76% of the 63 000 malaria deaths estimated in 2022 occurred in children aged under 5 years. The increasing prevalence of drug‐resistant *P. falciparum* strains and the poor availability of the RTS, S and R21/Matrix‐M vaccines in the endemic area led to an urgent need of novel antimalarial drugs.^[^ ^9^ ^]^

Leishmaniasis is caused by protozoa parasites, which are transmitted by the bite of infected female phlebotomine sandflies. The three main clinical forms of leishmaniases (i.e., visceral, cutaneous, and mucocutaneous) account for a total number of 12 million cases worldwide, with an estimated 700 000 to 1 million new cases every year.^[^ ^10^ ^]^ Over twenty Leishmania species caused leishmaniasis including *L.donovani* and *L.infatum* as major causative protozoa of visceral leishmaniasis and *L.tropica*, responsible for cutaneous leishmaniasis.^[^ ^11^, ^12^ ^]^ The current treatments for visceral and cutaneous leishmaniasis include pentavalent antimonials (i.e., sodium stibogluconate and meglumine antimoniate), miltefosine, amphotericin B, paromomycin, and azoles medicines (e.g., ketoconazole, fluconazole, itraconazole).^[^ ^13^ ^]^ However, the lack of drugs with novel mechanisms of action and the onset of drug‐resistance significantly reduce the effectiveness of current antiprotozoal therapies and call for novel effective compounds.

**Figure 1:**
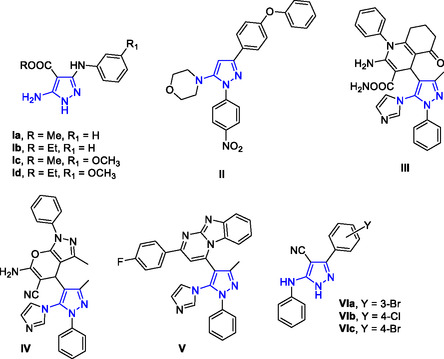
Pyrazole derivatives reported as antimalarial agents. The 5‐aminopyrazole substructure is colored blue.

In this scenario, amino‐pyrazoles (APs) represent a very useful and versatile scaffold for the synthesis of alternative antiprotozoal agents.^[^ ^14^ ^]^ In particular, 5‐amino pyrazoles (5‐APs) **Ia–d** (**Figure** **1**) exhibited sub‐micromolar IC_50_ values against *P. falciparum* in in vitro assays, being the methyl ester derivatives **Ia,c** significantly effective against *Leishmania donovani*.^[^ ^4^, ^5^ ^]^ 5‐Morpholine‐substituted pyrazole **II** (Figure 1) displayed promising antiprotozoal activity against *P. falciparum* chloroquine (CQ)‐resistant strain (IC_50_ = 3.7 μM) and *Leishmania donovani* (IC_50_ = 4.8 μM); additionally, the compound was not cytotoxic against normal myoblast L6 cells.^[^ ^15^ ^]^ More recently, 5‐imidazopyrazoles **III–V** (Figure 1) exhibited nanomolar activity against *P. falciparum* (IC_50_ = 30–35 nM) in vitro assays, resulting as active or more potent than CQ.^[^ ^16^ ^]^ Furthermore, compound **V** showed promising antibacterial properties with good inhibitory activity against a panel of Gram‐negative bacteria strains.^[^ ^17^ ^]^

Highly substituted APs **VI** (Figure 1) have been recently identified as novel antiplasmodial agents active against the CQ‐sensitive D10 and the CQ‐resistant W2 *P. falciparum* strains with IC_50_ values in the micromolar range. Additionally, the compounds were devoid of any cytotoxicity against normal fibroblasts.^[^ ^18^, ^19^ ^]^

**Figure 2:**
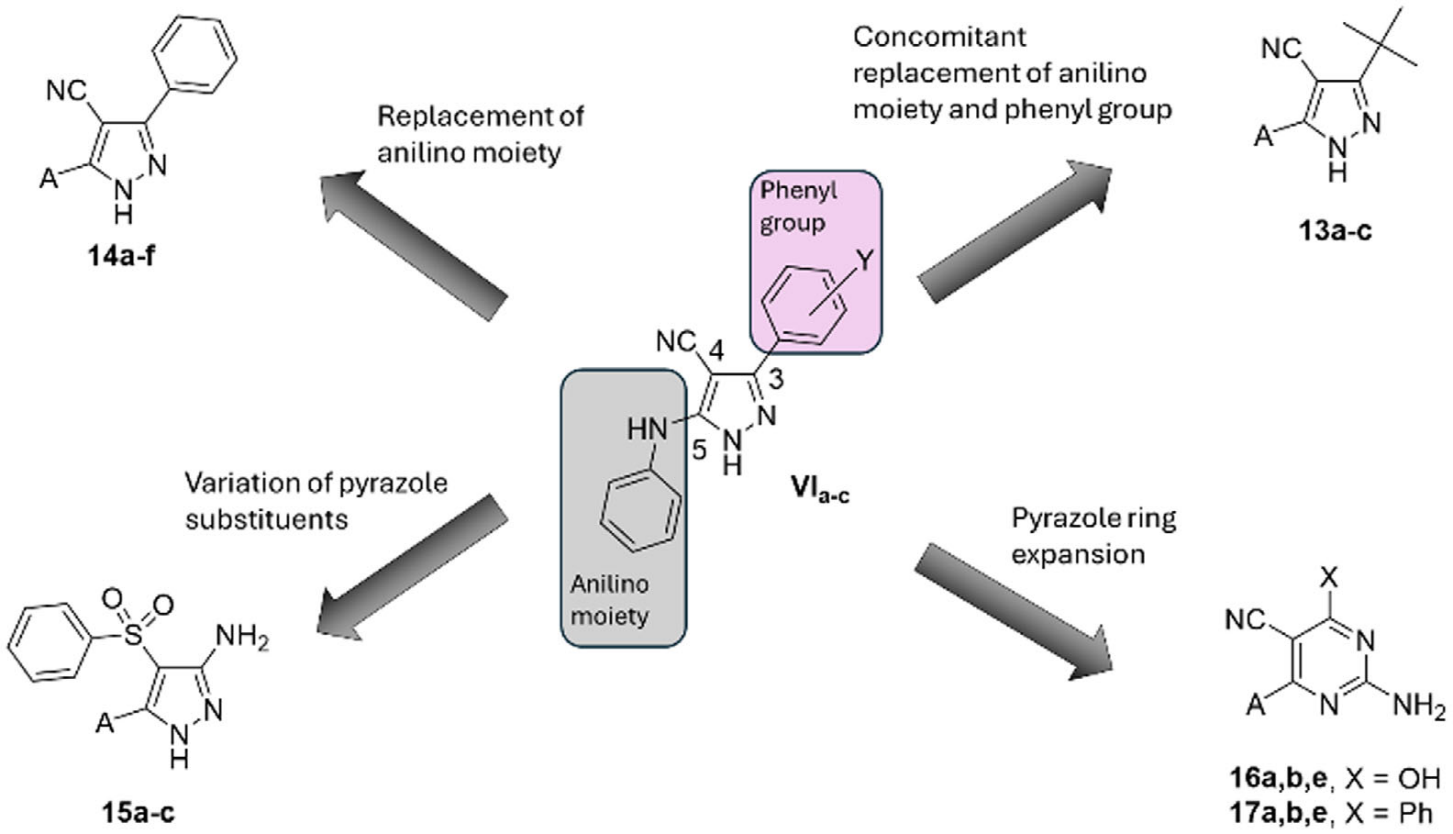
Modifications of lead compounds **VI**. The anilino moiety and the phenyl group are highlighted. The chemical identity of A substituents is reported in Scheme 1 and Table 1 and 2.

**Scheme 1:**
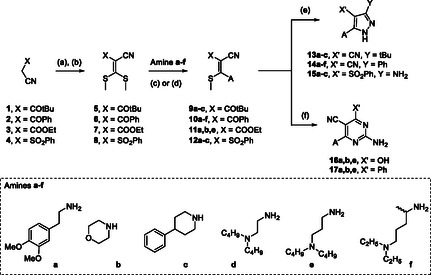
Synthesis of derivatives **13**–**17**. Reaction conditions: a) NaH, CS_2_, DMF_dry_, rt, 2 h. b) MeI, rt, 16 h. c) anhydrous Et_2_O, rt, 1 h. d) absolute EtOH, microwave 80 W, 5 min. e) EtOH, NH_2_NH_2_.H_2_O, rt, or reflux, 2 h. f) DMF_dry_, guanidine hydrochloride, K_2_CO_3_, 100 °C, 4 h. The A substituents are reported in Table 1.

To further extend the structure‐activity relationships (SARs) of pyrazoles **VI**, a novel series of derivatives (compounds **13–17**; **Figure** **2**) was designed and synthesized. In particular, trisubstituted pyrazoles **14** share with derivatives **VIa–c** the 3‐phenyl and the 4‐nitrile substituents on the pyrazole ring but bear different (aryl)alkyl and cycloalkyl chains replacing the anilino moiety at position 5. The concomitant replacement of **VI** anilino and phenyl moieties led to derivatives **13**, characterized by a hindered, aliphatic tert‐butyl group. In pyrazoles **15**, a free amino group and a phenylsulfonyl portion replaced the phenyl and the nitrile groups of **VI**, respectively. Finally, to evaluate the effect on antimalarial activity of the expansion of the central heterocyclic core, the pyrazole ring was replaced by a six‐membered pyrimidine scaffold (derivatives **16** and **17**; Figure 2), replacing (compounds **16**) or preserving (compounds **17**) the 3‐phenyl group of lead compounds **VI**. The substituents inserted to replace the anilino moiety of **VI** include arylalkyl (**a,c**; **Scheme** **1**), cycloalkyl (**b**; Scheme 1), and ω‐alkylamino (**d–f**; Scheme 1) chains characterized by different electronic, steric, and lipophilic properties.

## 2 Results and Discussion

### 2.1 Chemistry

The desired compounds **13–17** were prepared through a stepwise procedure starting from active methylene reagents (AMRs) **1–4** (Scheme 1).

Thus, the one‐pot condensation of AMRs with carbon disulfide in the presence of sodium hydride followed by in situ methylation with iodomethane led to the isolation of the ketene *S,S*‐acetals **5–8,** which were then reacted with amines **a–f** to afford compounds **9–12** (Scheme 1). According to the different reactivity of intermediates **5‐8** toward selected amines, two synthetic protocols were used to displace the S‐methyl group. Briefly, ketene *S,S*‐acetals **5** and **6** were reacted with amines **a–f** in anhydrous Et_2_O at rt to obtain compounds **9** and **10**, while the condensation of intermediates **7** and **8** with the proper amine in absolute ethanol, under microwave irradiation, led to the isolation of ketene *N,S*‐acetals **11** and **12**. It is worth mentioning that intermediates **9–12** can be classified as push–pull alkenes, that is, substituted alkenes bearing one or two electron‐donating groups (EDG) at one end of the double bond and one or two electron‐accepting groups at the other end.^[^ ^20^, ^21^ ^]^ This configuration promotes the π delocalization and the intramolecular charge transfer from the “push” terminus EDG to the “pull” terminus (electron‐withdrawing groups) affecting the molecular structure (e.g., central double bond elongation and rotational barrier of the unsaturated bond),^[^ ^22^, ^23^, ^24^ ^]^ the physicochemical properties (e.g., dipole moments, high hyperpolarizabilities^[^ ^25^, ^26^ ^]^ and the existence of strong change‐transfer absorption bands)^[^ ^27^ ^]^ and the pharmacological profiles (e.g., antitumor, anticonvulsant, and antibacterial)^[^ ^28^, ^29^, ^30^ ^]^ of the compounds. Push–pull intermediates **9–12** were finally cyclized with either hydrazine or guanidine to afford the desired pyrazole or pyrimidine compounds, respectively (Scheme 1). In particular, the reaction of **9**, **10,** and **12** with hydrazine monohydrate in absolute ethanol led to the isolation of pyrazoles **13–15,** whereas the condensation of **10** and **11** with guanidine hydrochloride in dry DMF at 100 °C afforded 2‐aminopyrimidines **16** and **17**. Noteworthy, compounds **9–11** bear two electrophilic centers (namely, a keto carbonyl and a nitrile group for **9** and **10** and an ester carbonyl and a nitrile group for **11**) potentially reactive with nucleophilic hydrazine and guanidine. This could lead to the possible formation of two different pyrazole and pyrimidine isomers. However, in the adopted conditions, the cyclization reaction proved to be highly chemo‐selective, resulting both the keto and the ester carbonyls being more reactive than nitrile toward the hydrazine/guanidine nucleophiles. Therefore, a single pyrazole and pyrimidine isomer has been isolated.

### 2.2 Antiprotozoal Activity and Cytotoxicity

The prepared pyrazoles and pyrimidine derivatives were evaluated for their antiplasmodial activity against CQ‐sensitive D10 and CQ‐resistant W2 *P. falciparum* strains. Additionally, derivatives **13–17** (and their lead congeners **VIa–c**) were tested for their antileishmanial properties against *L. tropica* and *L. infantum* promastigotes. CQ and amphotericin B (AmB) were used as reference drugs (**Table** **1** and **2**).

**Table 1:** Antiplasmodial and antileishmanial activity and cytotoxicity of pyrazole derivatives **13**–**15**.

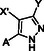								
Cpds	X′	Y	A	IC_50_ [μM]a)				Cell viability [%]a), b)
				*P. falciparum*				
				D10	W2	*L. infantum*	*L. tropica*	GM‐6114
13a	CN	*t*Bu	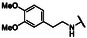	28.64	25.63	40.10	52.16	82
13b	CN	*t*Bu	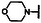	NA	NA	NA	NA	91
13c	CN	*t*Bu	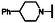	14.88	11.14	30.35	34.63	94
14a	CN	Ph	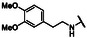	35.10	36.50	40.15	55.74	87
14b	CN	Ph	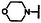	NA	NA	75.94	NA	84
14c	CN	Ph	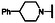	31.00	23.36	22.44	21.31	76
14 d	CN	Ph	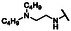	9.52	9.00	7.92	11.58	71
14e	CN	Ph	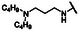	9.79	4.34	6.17	8.32	65
14f	CN	Ph	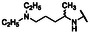	43.31	13.41	11.00	9.74	58
15a	SO_2_Ph	NH_2_	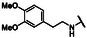	NA	NA	NA	NA	70
15b	SO_2_Ph	NH_2_	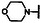	49.65	50.36	NA	NA	87
15c	SO_2_Ph	NH_2_	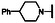	37.99	51.76	27.43	43.95	102
VIa	CN	(3‐Br)C_6_H_4_	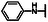	27.77	21.03	17.72	21.17	78
VIb	CN	(4‐Cl)C_6_H_4_	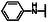	34.27	32.69	19.10	23.21	70
VIc	CN	(4‐Br)C_6_H_4_	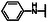	19.75	19.87	19.28	23.17	67
CQ				0.04	0.45	ND	ND	ND
AmB				ND	ND	0.22	0.29	ND

a) Data are the mean of three independent experiments run in duplicate;

b) Compounds were tested at 10 μM for 48 h. NA = not active. ND = not detected. CQ = CQ. AmB = amphotericin B;

**Table 2:** Antiplasmodial and antileishmanial activity and cytotoxicity of pyrimidine derivatives **16** and **17**.

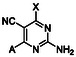							
			IC_50_ [μM]a)				Cell viability [%]a) ^,^ b)
			*P. falciparum*				
Cpd	X	A	D10	W2	*L. infantum*	*L. tropica*	GM‐6114
16a	OH	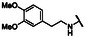	9.24	31.69	NA	NA	67
16b	OH	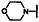	NA	NA	NA	NA	65
16e	OH	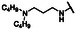	NA	44.95	NA	NA	66
17a	Ph	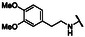	NA	NA	NA	NA	46
17b	Ph	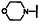	NA	NA	NA	NA	66
17e	Ph	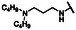	16.69	4.18	7.31	9.14	67
CQ			0.04	0.45	ND	ND	ND
AmB			ND	ND	0.22	0.29	ND

a) Data are the mean of three independent experiments run in duplicate;

b) Compounds were tested at 10 μM for 48 h. NA = not active. ND = not detected. CQ = CQ. AmB = amphotericin B;

Within the pyrazole series (derivatives **13–15**), the 3‐phenyl‐4‐nitrile derivative **14d** bearing a (dibutylamino)ethylamino portion showed similar activity against D10 and W2 strains and emerged more active than the lead compounds **VIa–c**. The elongation of the ω‐aminoalkyl chain did not significantly affect the activity against D10 strain but markedly increased the antiplasmodial properties against CQ‐resistant W2 strain (compare **14d** and **14e**). However, further chain elongation was detrimental for activity, being pyrazole **14f** less effective than its congeners **14d** and **14e** against both D10 and W2 strains. Despite this, **14e** and **14f** remained more effective against W2 strain compared to D10 strain. The insertion of a morpholine moiety at position 5 entirely abolished activity, whereas the insertion of aryl(cyclo)alkylamino substituents led to a significative reduction in antimalarial potency (compare **14a** and **14c** with **14d** and **14e**). The replacement of the 3‐phenyl substituent with a *ter*t‐butyl group marginally affected antiplasmodial activity when a 3,4‐dimethoxyphenethyl or a morpholino substituent is present (compare **13a** and **14a**; **13b,** and **14b**). However, compound **13c** showed improved antiplasmodial activity in comparison with its 3‐phenyl analog **14c**, sharing the same 4‐phenylpiperidin‐1‐yl chain. The insertion of 3‐amine‐4‐phenylsulfonyl‐substituents on the pyrazole nucleus proved to be detrimental for activity leading to inactive (**15a**) or poorly active (**15b** and **15c**) compounds.

Pyrazoles derivatives displayed a widespread micromolar antileishmanial activity, with derivatives **14d–f** showing single digit IC_50_ values against *L. tropica* and/or *L. infatum* protozoa, resulting more effective than their parent congeners **VIa–c** (Table 1). Interestingly, the three most active compounds share the 3‐phenyl‐4‐nitrile pyrazole scaffold and are characterized by differently hindered ω‐alkylamino chains at position 5.

The aminopyrimidine derivatives **16** and **17** displayed limited antiplasmodial activity with the exception of compounds **16a** and **17e** that showed micromolar IC_50_ values against D10 and W2 strains, respectively (Table 2). Interestingly, **17e** was found to be the sole pyrimidine analog active against *L. tropica* and *L. infatum* protozoa. Notably, both pyrazole **14e** and pyrimidine **17e** share the 3‐(dibutylamino)propyl)amino portion, which appeared to orient activity against CQ‐resistant W2 strain and *Leishmania* species.

Finally, with the unique exception of **17a,** all tested pyrazole (Table 1) and pyrimidine (Table 2) compounds proved to be noncytotoxic against human embryonic fibroblasts GM6114, showing cell viability percentage values higher than 50%.

### 2.3 Predicted Pharmacokinetic and Toxic Properties

To better characterize the pharmaceutical potentials of the prepared compounds, the calculated pharmacokinetic and toxic properties of derivatives **13–17** were predicted through the ADMETlab 3.0 platform.^[^ ^31^ ^]^ All prepared compounds were predicted to have favorable ADME profiles as defined by Lipinski (MW ≤ 500; logP ≤ 5; H‐bond acceptors ≤ 10; H‐bond donors ≤ 5) and golden triangle (200 ≤ MW ≤ 500; logD ≤ 5) rules.^[^ ^32^, ^33^ ^]^ As reported in Table S1 (Supporting Information), the majority of pyrazoles **13–15** (exception for **13a,b**) and pyrimidines **16**, **17** (exception for **16e**) would show intestinal absorption rate higher than 30% while derivatives **14a,b,** **15a–c,** **16a,b,** and **17b** would display bioavailability higher than 50%. Derivatives **13**, **14a,c,** and **17a,e** would inhibit *P*‐glycoprotein 1 (*P*‐gp) being only **13a** a substrate of this protein. The calculated plasma protein binding of the prepared compounds would be in the 37%–99% range and any compound would cross the blood brain barrier. Additionally, pyrazoles **13** and **14f** would inhibit organic anion transporters 1B1 and 1B3, whereas derivatives **14d,e,16e,** and **17e** would selectively block OATP1B3, thus influencing the distribution of these compounds. The distribution of all synthesized compounds would also be affected by their ability to inhibit Multidrug Resistance Protein 1 (MRP1) transporter without altering the activity of breast cancer resistance protein carrier. The various pyrazole and pyrimidine derivatives would differently interact with cytochrome P450 (CYP) isoforms 1A2, 2C19, 2C9, 2D6, 3A4, 2B6 and 2C8 acting as inhibitors and/or substrates (Table S2, Supporting Information). In particular, the morpholine derivatives **15b**, **16b,** and **17b** would not block the catalytic activity of any considered isoform, whereas the other derivatives would specifically inhibit various enzymes as indicated in Table S2 (Supporting Information). CYP 2C19 would represent the most affected isoform being inhibited by ten out of eighteen compounds. Conversely, CYP1A2 would be only inhibited by derivative **17a**. Most of the prepared compounds would be metabolized by CYPs 1A2 and 3A4, whereas **13b**, **14c**, **15c**, **16b,** and **17b** would not be recognized by any enzyme. With the exception of derivatives **15**, all compounds would show low (< 5 mL min^−1^ kg^−1^) or moderate (5–15 mL^−1^min kg^−1^) clearance and would present t_½_ values in the 0.5–1.1 h range.

The predicted toxicity profile (Table S3; Supporting Information) indicated that pyrazole derivatives **13–15** would show a probability higher than 70% to act as hERG blockers (**14c–f, 15,a,c**), liver injury inducers (**13a,b, 14a,b,d, 15a–c**), skin sensitizers (**14d–f, 15c**), carcinogens (**13b**), eye corrosives (**14e**) or irritants (**13a,b, 14a,b,e, 15b**), and respiratory toxicants (**13a–c, 14a–f**). Additionally, selected derivatives would induce hepatotoxic (**13b,c, 14c,f, 15a‐c**), nephrotoxic (**13b,c, 14b‐f, 15a,c**), ototoxic (**14f**), neurotoxic (**14a‐f**) and genotoxic (**13c, 14a,c, 15a–c**) effects. All derivatives would not be mutagenic nor hematotoxic and would not show acute oral toxicity. Finally, derivatives **14e,f** would be cytotoxic against human embryonic kidney cells HEK293 without affecting the growth of A549 human lung epithelial cells and Roswell Park Memorial Institute (RPMI)‐8226 lymphocytes.

Pyrimidines **16** and **17** would show neurotoxic (**16b,e, 17a,b,e**), genotoxic (**16b, 17a,b**), nephrotoxic (**16e, 17e**), hepatotoxic (**16b, 17a,b,e**), and carcinogenic (**16b**) effects. Additionally, selected derivatives would act as hERG blockers (**16e, 17e**), skin sensitizers (**16e, 17e**), eye irritants (**16a–c, 17b**), and respiratory toxicants (**16a,e, and 17e**). Finally, compounds **16a,b, 17a,b** would cause liver injury, but no pyrimidine compounds would show significative mutagenic, ototoxic, hematotoxic, eye corrosive, and oral acute toxic effects.

The analysis of potential toxicity pathways (Table S4; Supporting Information) suggested that aryl hydrocarbon nuclear receptor (NR‐AhR) pathway would mediate the toxicity of compounds **14a,f, 16e, 17a,e** whereas the stress response mitochondrial membrane potential (SR‐MMP) pathway would be involved in the toxicity of **13c, 14c,** **15c,** and **17a,b**. Aromatase pathway would be implied in the toxic effect of **14b**.

## 3 Conclusion

The SARs of antimalarial compounds **VIa–c** were extended through the synthesis of highly substituted pyrazole and pyrimidine derivatives **13–17**. The desired compounds were prepared according to a chemo‐selective divergent approach starting from *N,S*‐acetals **9–12** and tested for their antimalarial and antileishmanial activities. 3‐Phenyl‐4‐cyano pyrazoles **14d,e** were identified as the most promising derivatives of the series, showing micromolar activity against all considered protozoal species (i.e., *P. falciparum*, *L. infantum,* and *L. tropica).* Interestingly, the antimalarial profile of **14e** was mainly oriented against CQ‐resistant W2 *Plasmodium* strain and showed improved antiprotozoal efficacy in comparison with the lead compounds **VI**. The enlargement of the five‐membered pyrazole scaffold to a six‐membered pyrimidine nucleus allowed the identification of **17e**, the sole pyrimidine derivative effective against CQ‐sensitive D10 and CQ‐resistant W2 *Plasmodium* strains as well as against leishmanial species. Noteworthy, **14e** and **17e** share the 3‐(dibutylamino)propyl)amino portion and were devoid of any cytotoxicity against normal fibroblasts. The ADME predictions of these two derivatives indicated favorable pharmacokinetic properties in terms of Lipinski rules, intestinal absorption, and plasma protein binding. The two derivatives would share a common CYP profile with a predicted half life of 30 min. The predicted toxicity profile of **14e** and **17e** would include hERG inhibition, nephrotoxicity, respiratory toxicity, neurotoxicity, and skin sensibilization. Additionally, **17e** would display hepatoxicity, and **14e** would result an eye irritant and corrosive. Notably, the two compounds would not show carcinogenic, ototoxic, hematotoxic, mutagenic, and genotoxic effects. NR‐AhR pathway would mediate the toxicity of compound **17e** whereas pyrazole **14e** would not affect any toxicity pathways defined by Tox21 Consortium. Overall, the collected data led to the identification of novel antimalarial and antileishmanial agents and provide a foundation for further studies aimed at discovering more potent antiplasmodial pyrazole and pyrimidine compounds.

## 4 Experimental Section

4.1

4.1.1

##### Chemistry

Commercially available AMRs, amines, hydrazine hydrate, guanidine hydrochloride, and reagents (55% sodium hydride dispersion in mineral oil, iodomethane, and carbon disulfide) were purchased from Alfa–Aesar and Sigma–Aldrich. DMF was reagent grade and was dried on molecular sieves (5 Å 1/16" inch pellets). Unless otherwise stated, all commercial reagents were used without further purification. Organic solutions were dried over anhydrous sodium sulphate. Thin‐layer chromatography system for routine monitoring of the course of parallel reactions and confirming the purity of analytical samples employed aluminum‐backed silica gel plates (Merck DC‐Alufolien Kieselgel 60 F254). DCM or DCM/methanol (9:1) were used as a developing solvent, and detection of spots was made by UV light and/or by iodine vapors. Microwave irradiation was carried out by a Prolabo Synthewave 402 instrument. Melting points were determined on a Fisher‐Johns apparatus and are uncorrected. ^1^H NMR and ^13^C NMR spectra were recorded on a JEOL JNM‐ECZR instrument; chemical shifts were reported in δ (ppm) units relative to the internal reference tetramethylsilane, and the splitting patterns were described as follows: s (singlet), bs (broad singlet), d (doublet), t (triplet), q (quartet), and m (multiplet). The first‐order values reported for coupling constants *J* were given in Hz. Elemental analyzes were performed by an EA1110 Analyzer, Fison instruments (Milan).

##### General Synthetic Procedure for the Preparation of Ketene S,S‐Acetals 5‐8

To a dry DMF solution (10 mL) of the proper AMR (10 mmol), 55% sodium hydride dispersion in mineral oil (0.44 g, 10 mmol) and CS_2_ (607 μL, 10 mmol) were sequentially added under stirring at 0 °C. The mixture was stirred at rt for 2 h and then iodomethane (1258 μL, 20 mmol) was added, prolonging stirring at rt for 16 h. The reaction mixture was cooled, treated with water (50 mL), and the precipitated solid was collected by filtration, dried, and used without further purification. For compound **5**, the water solution was extracted with DCM (2 × 20 mL), and the pooled organic phases were washed with water (5 × 10 mL) and dried with Na_2_SO_4_. After evaporating the solvent in vacuo, the crude material was purified by distillation.

*2‐(bis(methylthio)methylene)‐4,4‐dimethyl‐3‐oxopentanenitrile* *(**5**)*. Yellow oil. Bp 100 °C/3 mmHg (Litt.: 148–149 °C);^[^ ^34^ ^]^ Yield: 41%.

*2‐benzoyl‐3,3‐bis(methylthio)acrylonitrile* *(**6**)*. Mp 65–69 °C (H_2_O) (Litt.: 71–72 °C);^[^ ^35^ ^]^ Yield: 95%.

*ethyl 2‐cyano‐3,3‐bis(methylthio)acrylate (**7**)*. Mp 55–57 °C (H_2_O) (Litt.: 56–57 °C);^[^ ^36^ ^]^ Yield: 88%

*3,3‐bis(methylthio)‐2‐(phenylsulfonyl)acrylonitrile (**8**)*. Mp 83–86 °C (H_2_O) (Litt.: 102–103 °C);^[^ ^37^ ^]^ Yield: 86%.

##### Synthesis of Intermediates 9 and 10

An anhydrous Et_2_O solution (15 mL) of intermediate **5** or **6** (3 mmol) and the proper amine **a–f** (3 mmol) was stirred at rt for 1 h. For compounds **9** and **10d–f**, the solvent was evaporated in vacuo, and the crude material was used in the next step without further purification. For compounds **10a–c**, the precipitated solid was collected by filtration, dried, and used without further purification.

*4,4‐dimethyl‐2‐(((3,4‐dimethoxyphenethyl)amino)(methylthio)methylene)‐3‐oxopentanenitrile (**9a**)*. Yellow oil. Calcd for C_19_H_26_N_2_O_3_S: C = 62.96; H = 7.23; N = 7.73; S = 8.84. Found: C = 63.25; H = 7.12; N = 7.43; S = 8.81.

*4,4‐dimethyl‐2‐((methylthio)(morpholino)methylene)‐3‐oxopentanenitrile (**9b**)*. Yellow oil. Calcd for C_13_H_20_N_2_O_2_S: C = 58.18; H = 7.51; N = 10.44; S = 11.95. Found: C = 58.04; H = 7.57; N = 7.62; S = 11.84.

*4,4‐dimethyl‐2‐((methylthio)(4‐phenylpiperidin‐1‐yl)methylene)‐3‐oxopentanenitrile (**9c**)*. Yellow oil. Calcd for C_20_H_26_N_2_OS: C = 70.14; H = 7.65; N = 8.18; S = 9.36. Found: C = 70.34; H = 7.75; N = 8. 62; S = 9.54.

*2‐benzoyl‐3‐((3,4‐dimethoxyphenethyl)amino)‐3‐(methylthio)acrylonitrile (**10a**)*. Mp 73–74 °C (Et_2_O); Yield: 63%. Calcd for C_21_H_22_N_2_O_3_S: C = 65.95; H = 5.80; N = 7.32; S = 8.38. Found: C = 65.70; H = 5.74; N = 7.08; S = 8.69.

*2‐benzoyl‐3‐(methylthio)‐3‐morpholinoacrylonitrile (**10b**)*. Mp 130–135 °C (Et_2_O); Yield: 54%. Calcd for C_15_H_16_N_2_O_2_S: C = 62.48; H = 5.59; N = 9.71; S = 11.12. Found: C = 62.28; H = 5.48; N = 9.74; S = 11.00.

*2‐benzoyl‐3‐(methylthio)‐3‐(4‐phenylpiperidin‐1‐yl)acrylonitrile (**10c**)*. Mp 144–‐146 °C (Et_2_O); Yield: 83%. Calcd for C_22_H_22_N_2_OS: C = 72.90; H = 6.12; N = 7.73; S = 8.84. Found: C = 72.45; H = 6.34; N = 7.93; S = 8.51.

*2‐benzoyl‐3‐((2‐(dibutylamino)ethyl)amino)‐3‐(methylthio)acrylonitrile (**10d**)*. Brown oil. Calcd for C_21_H_31_N_3_OS: C = 67.52; H = 8.36; N = 11.25; S = 8.58. Found: C = 67.27; H = 8.14; N = 11.53; S = 8.76.

*2‐benzoyl‐3‐((3‐(dibutylamino)propyl)amino)‐3‐(methylthio)acrylonitrile (**10e**).* Brown oil. Calcd for C_22_H_33_N_3_OS: C = 68.18; H = 8.58; N = 10.84; S = 8.27. Found: C = 68.37; H = 8.77; N = 10.67; S = 8.46.

*2‐benzoyl‐3‐((5‐(diethylamino)pentan‐2‐yl)amino)‐3‐(methylthio)acrylonitrile (**10f**)*. Brown oil. Calcd for C_20_H_29_N_3_OS: C = 66.81; H = 8.13; N = 11.69; S = 8.92. Found: C = 66.51; H = 8.29; N = 12.01; S = 9.11.

##### Synthesis of Intermediates 11 and 12

An absolute ethanol solution (5 mL) of intermediate **7** or **8** (3 mmol) and the proper amine **a–** **c** or **e** (3 mmol) was stirred for 5 min under microwave irradiation (80 Watt). The precipitated solid was collected by filtration and used without further purification.

*ethyl 2‐cyano‐3‐((3,4‐dimethoxyphenethyl)amino)‐3‐(methylthio)acrylate (**11a**).* Mp 92–95 °C (EtOH); Yield: 68%. Calcd for C_17_H_22_N_2_O_4_S: C = 58.27; H = 6.33; N = 7.99; S = 9.15. Found: C = 58.09; H = 6.23; N = 8.14; S = 8.89.

*ethyl 2‐cyano‐3‐(methylthio)‐3‐morpholinoacrylate (**11b**).* Mp 95–97 °C (EtOH); Yield: 82%. Calcd for C_11_H_16_N_2_O_3_S: C = 51.55; H = 6.29; N = 10.93; S = 12.51. Found: C = 51.57; H = 6.52; N = 11.04; S = 12.36.

*ethyl 2‐cyano‐3‐((3‐(dibutylamino)propyl)amino)‐3‐(methylthio)acrylate (**11e**).* Yellow oil. Yield: 73%. Calcd for C_18_H_33_N_3_O_2_S: C = 60.81; H = 9.36; N = 11.82; S = 9.02. Found: C = 60.90; H = 9.23; N = 12.14; S = 8.98.

*3‐((3,4‐dimethoxyphenethyl)amino)‐3‐(methylthio)‐2‐(phenylsulfonyl)acrylonitrile* *(**12a**)*. Mp 126–129 °C (EtOH); Yield: 72%. ^1^H NMR (400 MHz, CDCl_3_): δ 2.48 (s, 3H, SCH_3_); 2.89 (t, 2H, *J* = 6.7Hz, CH_2_Ph); 3.78–3.84 (m, 2H, CH_2_N); 3.87 (s, 3H, OCH_3_); 3.88 (s, 3H, OCH_3_); 6.73–6.79 (m, 2H, arom. H); 6.83–6.86 (m, 1H, arom. H); 7.43–7.49 (m, 2H, arom. H); 7.56–7.61 (m, 1H, arom. H); 7.65–7.69 (m, 2H, arom. H); 8.55 (bs, 1H, NH, exchangeable). Calcd for C_20_H_22_N_2_O_4_S_2_: C = 57.40; H = 5.30; N = 6.69; S = 15.32. Found: C = 57.55; H = 5.31; N = 6.87; S = 15.22.

*3‐(methylthio)‐3‐morpholino‐2‐(phenylsulfonyl)acrylonitrile (**12b**)*. Mp 95–98 °C (EtOH); Yield: 70%. ^1^H NMR (400 MHz, CDCl_3_): δ 2.44 (s, 3H, SCH_3_); 3.77–3.80 (m, 4H, 2xCH_2_ morph.); 3.84–3.87 (m, 4H, 2xCH_2_ morph.); 7.51–7.60 (m, 3H, arom. H); 7.93–7.95 (m, 2H, arom. H). Calcd for C_14_H_16_N_2_O_3_S_2_: C = 51.83; H = 4.97; N = 8.64; S = 19.76. Found: C = 52.11; H = 5.08; N = 8.24; S = 19.56.

*3‐(methylthio)‐3‐(4‐phenylpiperidin‐1‐yl)‐2‐(phenylsulfonyl)acrylonitrile (**12c**)*. Mp 145–147 °C (EtOH); Yield: 73%. ^1^H NMR (400 MHz, CDCl_3_): δ 1.76–1.87 (m, 2H, CH_2_ pip.); 2.00–2.04 (m, 2H, CH_2_ pip.); 2.48 (s, 3H, SCH_3_); 2.87–2.94 (m, 1H, CH pip.); 3.53–3.60 (m, 2H, CH_2_N pip.); 4.32–4.36 (m, 2H, CH_2_N pip.); 7.19–7.35 (m, 5H, arom. H); 7.51–7.61 (m, 3H, arom. H); 7.96–7.99 (m, 2H, arom. H). Calcd for C_21_H_22_N_2_O_2_S_2_: C = 63.29; H = 5.56; N = 7.03; S = 16.09. Found: C = 63.57; H = 5.55; N = 7.39; S = 16.20.

##### General Synthetic Procedure for the Preparation of Pyrazoles 13‐15

To an absolute ethanol solution (10 mL) of the proper *S*‐methyl intermediate **9**, **10,** or **12** (1 mmol), hydrazine monohydrate (54 μL, 1.1 mmol), was added and the reaction mixture was stirred at rt for 30 min (compounds **13** and **14**) or refluxed for 2 h (compounds **15**). After cooling at rt the solvent was concentrated in vacuo. The crude compounds were purified either by column chromatography (derivatives **13**; silica gel; DCM–DCM 4%MeOH gradient) or by crystallization from the proper solvent (compounds **14** and **15**).

*3‐(tert‐butyl)‐5‐((3,4‐dimethoxyphenethyl)amino)‐1H‐pyrazole‐4‐carbonitrile (**13a**)*.Yellow oil. Yield: 38%. ^1^H NMR (400 MHz, DMSO‐d_6_): δ 1.28 (s, 9H, 3xCH_3_); 2.73–2.77 (m, 2H, CH_2_Ph); 3.27–3.31 (m, 2H, CH_2_N); 3.71(s, 3H, OCH_3_); 3.73 (s, 3H, OCH_3_); 4.24 (bs, 1H, NH, exchangeable); 6.73–6.75 (m, 1H, arom. H); 6.83–6.86 (m, 2H, arom. H). ^13^C NMR (101 MHz, DMSO‐d_6_): δ 162.35; 148.61; 147.23; 131.96; 120.55; 116.56; 112.65; 111.90; 55.54; 55.36; 44.87; 34.78. Calcd for C_18_H_24_N_4_O_2_: C = 65.83; H = 7.37; N = 17.06. Found: C = 65.76; H = 7.23; N = 16.89.

*3‐(tert‐butyl)‐5‐morpholino‐1H‐pyrazole‐4‐carbonitrile (**13b**)*. Mp 136–138 °C; Yield: 35%. ^1^H NMR (400 MHz, DMSO‐d_6_): δ 1.34 (s, 9H, 3xCH_3_); 2.49–2.51 (m, 4H, 2xCH_2_N morph.); 3.69–3.71 (m, 4H, CH_2_O morph.); 12.52 (bs, 1H, NH, exchangeable). ^13^C NMR (101 MHz, DMSO‐d_6_): δ 160.18; 158.29; 116.35; 75.42; 65.62; 48.32; 32.23; 28.69 Calcd for C_12_H_18_N_4_O: C = 61.52; H = 7.74; N = 23.91. Found: C = 61.19; H = 7.52; N = 24.25.

*3‐(tert‐butyl)‐5‐(4‐phenylpiperidin‐1‐yl)‐1H‐pyrazole‐4‐carbonitrile* *(**13c**)*. Mp 112–116 °C; Yield: 21%. ^1^H NMR (400 MHz, CDCl_3_): δ 1.46 (s, 9H, 3xCH_3_); 1.80–1.96 (m, 4H, 2xCH_2_ pip.); 2.64–2.73 (m, 1H, CH pip.); 3.02–3.09 (m, 2H, CH_2_N pip.); 4.16–4.20 (m, 2H, CH_2_N pip.); 7.19–7.22 (m, 3H, arom. H); 7.28–7.32 (m, 2H, arom. H). ^13^C NMR (101 MHz, CDCl_3_): δ 161.22; 157.99; 145.23; 128.73; 126.87; 126.69; 115.54; 76.36; 49.25; 42.11; 33.22; 32.73. Calcd for C_19_H_24_N_4_: C = 73.99; H = 7.84; N = 18.17. Found: C = 73.68; H = 7.66; N = 17.91.

*5‐((3,4‐dimethoxyphenethyl)amino)‐3‐phenyl‐1H‐pyrazole‐4‐carbonitrile (**14a**)*. Mp 170–175 °C (EtOH); Yield: 77%. ^1^H NMR (400 MHz, DMSO‐d_6_): δ 2.79–2.82 (m, 2H, CH_2_Ph); 3.36–3.41 (m, 2H, CH_2_N); 3.71 (s, 3H, OCH_3_); 3.75 (s, 3H, OCH_3_); 6.76–6.89 (m, 4H, arom. H + NH, exchangeable); 7.40–7.51 (m, 3H, arom. H); 7.78–7.81 (m, 2H, arom. H); 12.33 (bs, 1H, NH pyraz., exchangeable). ^13^C NMR (101 MHz, DMSO‐d_6_): δ 155.84; 148.62; 147.28; 131.74; 129.02; 128.88; 125.87; 120.61; 116.36; 112.70; 111.91; 70.06; 55.53; 55.37; 44.91; 34.77. Clacd for C_20_H_20_N_4_O_2_: C = 68.95; H = 5.79; N = 16.08. Found: C = 65.87; H = 5.76; N = 15.89.

*5‐morpholino‐3‐phenyl‐1H‐pyrazole‐4‐carbonitrile (**14b**).* Mp 157–162 °C (EtOH); Yield: 55%. ^1^H NMR (400 MHz, DMSO‐d_6_): δ 3.31–3.37 (m, 4H, 2xCH_2_N morph.); 3.73–3.76 (m, 4H, 2xCH_2_O morph.); 7.50–7.56 (m, 3H, arom H.); 7.76–7.78 (m, 2H, arom H.). ^13^C NMR (101 MHz, DMSO‐d_6_): δ 160.17; 147.70; 130.17; 129.16; 126.37; 116.21; 65.49; 48.04. Calcd for C_14_H_14_N_4_O: C = 66.13; H = 5.55; N = 22.03. Found: C = 65.85; H = 5.18; N = 21.89.

*3‐phenyl‐5‐(4‐phenylpiperidin‐1‐yl)‐1H‐pyrazole‐4‐carbonitrile (**14c**)*. Mp 179–180 °C (EtOH); Yield: 45%. ^1^H NMR (400 MHz, DMSO‐d_6_): δ 1.72–1.89 (m, 4H, 2xCH2 pip.); 2.71–2.77 (m, 1H, CH pip.); 3.01–3.07 (m, 2H, CH_2_N pip.); 4.01–4.04 (m, 2H, CH_2_N pip.); 7.19–7.31 (m, 5H, arom. H); 7.47–7.55 (m, 3H, arom. H); 7.78–7.81 (m, 2H, arom. H). ^13^C NMR (101 MHz, DMSO‐d_6_): δ 32.63; 41.73; 49.09; 117.26; 126.76; 126.83; 127.28; 128.97; 129.57; 130.04; 146.23. Calcd for C_21_H_20_N_4_: C = 76.80; H = 6.14; N = 17.06. Found: C = 76.51; H = 5.98; N = 16.94.

*5‐((2‐(dibutylamino)ethyl)amino)‐3‐phenyl‐1H‐pyrazole‐4‐carbonitrile (**14 d**).* Mp 150–153 °C (Et_2_O); Yield: 48%. ^1^H NMR (400 MHz, DMSO‐d_6_): δ 0.90 (t, 6H, *J* = 7.4Hz, 2xCH_3_); 1.26–1.37 (m, 4H, 2xCH_2_); 1.59–1.70 (m, 4H, 2xCH_2_); 3.03–3.13 (m, 4H, 2xCH_2_N); 3.23–3.29 (m, 2H, CH_2_N); 3.50–3.63 (m, 2H, CH_2_N); 6.42 (bs, 1H, NH, exchangeable); 7.39–7.60 (m, 3H, arom. H); 7.76–7.81 (m, 2H, arom. H); 10.36 (bs, 1H, NH pyraz., exchangeable). ^13^C NMR (101 MHz, DMSO‐d_6_): δ 158.13; 146.46; 130.11; 129.29; 126.08; 124.33; 99.41; 83.75; 52.18; 50.04; 37.61; 24.86; 19.47; 13.58. Calcd for C_20_H_29_N_5_: C = 70.76; H = 8.61; N = 20.63. Found: C = 70.71; H = 8.59; N = 20.43.

*5‐((3‐(dibutylamino)propyl)amino)‐3‐phenyl‐1H‐pyrazole‐4‐carbonitrile* *(**14e**)*. Mp 160–162 °C (EtOH); Yield: 37%. ^1^H NMR (400 MHz, DMSO‐d_6_): δ 0.89 (t, 6H, *J* = 7.2Hz, 2xCH_3_); 1.22–1.37 (m, 4H, 2xCH_2_); 1.55–1.68 (m, 4H, 2xCH_2_); 1.89–2.00 (m, 2H, CH_2_); 2.92–3.03 (m, 4H, 2xCH_2_N); 3.05–3.13 (m, 2H, CH_2_N); 3.20–3.20 (m, 2H, CH_2_N); 7.16 (bs, 1H, NH, exchangeable); 7.36–7.58 (m, 3H, arom. H); 7.75–7.83 (m, 2H, arom. H); 10.54 (bs, 1H, NH pyraz., exchangeable). ^13^C NMR (101 MHz, DMSO‐d_6_): δ 154.24; 128.93; 125.90, 116.20; 83.98; 51.64; 49.26; 24.90; 22.77; 19.50; 13.56. Calcd for C_21_H_31_N_5_: C = 71.35; H = 8.84; N = 19.81. Found: C = 71.28; H = 8.72; N = 19.73.

*5‐((5‐(diethylamino)pentan‐2‐yl)amino)‐3‐phenyl‐1H‐pyrazole‐4‐carbonitrile (**14f**)*. Mp 127–129 °C (Et_2_O/Ligroin); Yield: 53%. ^1^H NMR (400 MHz, DMSO‐d_6_): δ 0.93 (t, 6H, *J* = 7.1Hz, 2xCH_3_); 1.13–1.17 (d, 2H, *J* = 6.4Hz, CH_3_CH); 1.39–1.59 (m, 4H, 2xCH_2_); 2.32–2.47 (m, 6H, 3xCH_2_N); 3.49–3.59 (m, 1H, CHN); 6.63 (m, 1H, NH, exchangeable); 7.39–7.53 (m, 3H, arom. H); 7.73–7.82 (m, 2H, arom. H); 12.45 (bs, 1H, NH pyraz., exchangeable). ^13^C NMR (101 MHz, DMSO‐d_6_): δ 155.75; 149.96; 131.46; 129.34; 126.38; 117.04; 70.26; 52.49; 49.32; 46.67; 34.53; 23.71; 21.25; 12.03. Calcd for C_19_H_27_N_5_: C = 70.12; H = 8.36; N = 21.52. Found: C = 69.92; H = 8.26; N = 21.69.

*N* ^*5*^ *‐(3,4‐dimethoxyphenethyl)‐4‐(phenylsulfonyl)‐1H‐pyrazole‐3,5‐diamine (**15a**).* Mp 78–81 °C (EtOH); Yield: 73%. ^1^H NMR (400 MHz, DMSO‐d_6_): δ 2.74–2.77 (m, 2H, CH_2_Ph); 3.34–3.38 (m, 2H, CH_2_N); 3.73 (s, 3H, OCH_3_); 3.73 (s, 3H, OCH_3_); 4.92 (bs, 2H, NH_2_, exchangeable); 5.89 (bs, 1H, NH, exchangeable); 6.73–6.89 (m, 3H, arom. H); 7.48–7.61 (m, 3H, arom. H); 7.77–7.79 (m, 2H, arom. H); 10.86 (bs, 1H, NH pyraz., exchangeable). ^13^C NMR (101 MHz, DMSO‐d_6_): δ 148.67; 147.24; 144.66; 132.40; 129.09; 125.02; 120.56; 112.56; 111.88; 85.93; 56.06; 55.32; 43.75; 34.54. Calcd for C_19_H_22_N_4_O_4_S: C = 56.70; H = 5.51; N = 13.92; S = 7.97. Found: C = 56.36; H = 5.81; N = 13.57; S = 7.62.

*5‐morpholino‐4‐(phenylsulfonyl)‐1H‐pyrazol‐3‐amine (**15b**)*. Mp 186–188 °C (EtOH); Yield: 86%. ^1^H NMR (400 MHz, DMSO‐d_6_): δ 2.99–3.01 (m, 4H, 2xCH_2_N morph.); 3.60–3.62 (m, 4H, 2xCH_2_O morph.); 6.03 (bs, 2H, NH_2_, exchangeable); 7.54–7.63 (m, 3H, arom. H); 7.86–7.89 (m, 2H, arom. H); 11.23 (bs, 1H, NH, exchangeable). ^13^C NMR (101 MHz, DMSO‐d_6_): δ 155.50; 150.78; 144.32; 132.59; 129.04; 125.52; 90.22; 66.16; 51.10. Calcd for C_13_H_16_N_4_O_3_S: C = 50.64; H = 5.23; N = 18.17; S = 10.40. Found: C = 50.94; H = 5.28; N = 18.46; S = 10.68.

*5‐(4‐phenylpiperidin‐1‐yl)‐4‐(phenylsulfonyl)‐1H‐pyrazol‐3‐amine(**15c**)*. Mp 218–220 °C (EtOH); Yield: 88%. ^1^H NMR (400 MHz, DMSO‐d_6_): δ 1.58–1.75 (m, 4H, 2xCH_2_ pip.); 2.55–2.69 (m, 3H, CH pip + CH_2_N pip.); 3.56–3.59 (m, 2H, CH_2_N pip.); 5.98 (bs, 2H, NH_2_, exchangeable); 7.17–7.33 (m, 5H, arom. H); 7.56–7.63 (m, 3H, arom. H); 7.89–7.91 (m, 2H, arom. H); 11.36 (bs, 1H, NH pyraz., exchangeable). ^13^C NMR (101 MHz, DMSO‐d_6_): δ 146.16; 144.41; 132.48; 128.94; 128.42; 126.69; 126.08; 125.53; 90.15; 51.36; 41.56; 32.95. Calcd. for C_20_H_22_N_4_O_2_S: C = 62.81; H = 5.80; N = 14.65; S = 8.38. Found: C = 62.57; H = 5.67; N = 14.79; S = 8.45.

##### General Synthetic Procedure for the Preparation of Pyrimidines 16 and 17

In a sealed tube, few drops of DMF were added to a mixture of guanidine hydrochloride (184 mg, 1 mmol), K_2_CO_3_ (279 mg, 2 mmol) and the proper *S*‐methyl intermediate **10** or **11** (1 mmol). The reaction was heated at 100 °C for 4 h and then cooled at rt. The mixture was diluted with water (10 mL) and neutralized with HCl 2M. The precipitated solid was collected by filtration and recrystallized from the proper solvent or solvent mixture. Compound **17e** was purified by column chromatography (silica gel, eluent: AcOEt/20% EtOH).

*2‐amino‐4‐((3,4‐dimethoxyphenethyl)amino)‐6‐hydroxypyrimidine‐5‐carbonitrile (**16a***). Mp 217–220 °C (H_2_O); Yield: 58%. ^1^H NMR (400 MHz, DMSO‐d_6_) δ 2.68–2.79 (m, 2H, CH_2_Ar); 3.47–3.56 (m, 2H, CH_2_N); 3.71 (s, 3H, OCH_3_); 3.74 (s, 3H, OCH_3_); 6.66–6.98 (m, 5H, arom. H + NH_2_ exchangeable); 7.15–7.25 (m, 1H, NH, exchangeable); 10.43 (bs, 1H, OH, exchangeable). ^13^C NMR (101 MHz, DMSO‐d_6_) δ 164.69; 162.00; 155.76; 148.59; 147.23; 131.80; 120.52; 117.66; 112.56; 111.89; 63.93; 55.52; 55.37; 42.21; 34.85. Calcd. for C_15_H_17_N_5_O_3_: C = 57.13; H = 5.43; N = 22.21. Found: C = 56.90; H = 5.23; N = 22.14.

*2‐amino‐4‐hydroxy‐6‐morpholinopyrimidine‐5‐carbonitrile* *(**16b**). Mp* 250–253 °C (H_2_O); Yield: 63%. ^1^H NMR (400 MHz, DMSO‐d_6_) δ 3.57–3.68 (m, 4H, 2xCH_2_N); 3.70–3.82 (m, 4H, 2xCH_2_O); 7.14 (bs, 2H, NH_2_, exchangeable); 11.03 (bs, 1H, OH, exchangeable). ^13^C NMR (101 MHz, DMSO‐d_6_) δ 164.53; 154.98; 130.87; 119.08; 66.02; 46.66. Calcd. for C_9_H_11_N_5_O_2_: C = 48.86; H = 5.01; N = 31.66. Found: C = 48.72; H = 4.90; N = 31.62.

*2‐amino‐4‐((3‐(dibutylamino)propyl)amino)‐6‐hydroxypyrimidine‐5‐carbonitrile (**16e**).* Mp 74–76 °C (Et_2_O); Yield: 14%. ^1^H NMR (400 MHz, DMSO‐d_6_) δ 0.86 (t, *J* = 7.3Hz, 6H, 2xCH_3_); 1.19–1.41 (m, 8H, 4xCH_2_); 1.57–1.66 (m, 2H, CH_2_); 2.31–2.44 (m, 4H, 2xCH_2_N); 3.28–3.38 (m,  H, 2xCH_2_N); 6.97 (bs, 2H, NH_2_, exchangeable); 7.36–7.45 (m, 1H, NH, exchangeable); 10.45 (bs, 1H, OH, exchangeable). ^13^C NMR (101 MHz, DMSO‐d_6_) δ 164.67; 162.08; 155.85; 117.67; 63.83; 53.18; 51.48; 28.42; 26.04; 20.15; 13.95. Calcd. for C_16_H_28_N_6_O: C = 59.97; H = 8.81; N = 26.23. Found: C = 59.79; H = 8.78; N = 26.42.

*2‐amino‐4‐((3,4‐dimethoxyphenethyl)amino)‐6‐phenylpyrimidine‐5‐carbonitrile (**17a**).* Mp 227‐230 °C (H_2_O); Yield: 76%. ^1^H NMR (400 MHz, DMSO‐d_6_) δ 2.77–2.85 (m, 2H, CH_2_); 3.54–3.62 (m, 2H, CH_2_N); 3.71 (s, 3H, OCH_3_); 3.75 (s, 3H, OCH_3_); 6.74–6.78 (m, 1H, arom. H); 6.81–6.88 (m, 3H, arom. H); 7.22 (bs, 2H, NH_2_, exchangeable); 7.29–7.36 (m, 1H, arom. H); 7.45–7.55 (m, 3H, arom. H and NH exchangeable); 7.71–7.76 (m, 2H, arom. H). ^13^C NMR (101 MHz, DMSO‐d_6_) δ 169.09; 163.03; 162.77; 148.61; 147.24; 137.10; 131.91; 130.36; 128.27; 128.23; 120.56; 117.95; 112.53; 111.88; 76.64; 55.52; 55.38; 42.04; 34.31. Calcd. for C_21_H_21_N_5_O_2_: C = 67.18; H = 5.64; N = 18.65. Found: C = 67.41; H = 5.43; N = 18.37.

*2‐amino‐4‐morpholino‐6‐phenylpyrimidine‐5‐carbonitrile (**17b**).* Mp 173–174 °C (H_2_O); Yield: 51%. ^1^H NMR (400 MHz, DMSO‐d_6_) δ 3.65–3.72 (m, 4H, 2xCH_2_N); 3.75–3.82 (m, 4H, 2xCH_2_O); 7.38 (bs, 2H, NH_2_, exchangeable); 7.45–7.56 (m, 3H, arom. H); 7.72–7.77 (m, 2H, arom. H). ^13^C NMR (101 MHz, DMSO‐d_6_) δ 171.72; 164.61; 161.86; 136.83; 130.65; 128.88; 128.23; 119.27; 77.88; 65.96; 47.06. Calcd. for C_15_H_15_N_5_O: C = 64.04; H = 5.37; N = 24.90. Found: C = 64.19; H = 5.34; N = 24.77.

*2‐amino‐4‐((3‐(dibutylamino)propyl)amino)‐6‐phenylpyrimidine‐5‐carbonitrile (**17e**).* Mp 107–108 °C (Et_2_O); Yield: 12%. ^1^H NMR (400 MHz, DMSO‐d_6_) δ 0.89 (t, *J* = 7.2Hz, 6H, 2xCH_3_); 1.18–1.60 (m, 8H, 4xCH_2_); 1.72–1.96 (m, 2H, CH_2_); 2.69–2.99 (m, 4H, 2xCH_2_N); 3.38–3.47 (m, 4H, 2xCH_2_N); 7.07 (bs, 1H, NH, exchangeable); 7.26 (bs, 2H, NH_2_, exchangeable); 7.43–7.56 (m, 3H, arom. H); 7.68–7.77 (m, 2H, arom. H). ^13^C NMR (101 MHz, DMSO‐d_6_) δ 169.24; 163.21; 162.87; 137.15; 130.36; 128.26; 128.17; 117.96; 76.61; 56.09; 52.23; 30.37; 26.55; 19.65; 13.75. Calcd. for C_22_H_32_N_6_: C = 69.44; H = 8.48; N = 22.08. Found: C = 69.21, H = 8.30, N = 21.72.

##### Biology

**MTT assays:** MTT assays were performed using GM‐6114 (embryonic human fibroblast, ATCC, Manassas, VA). The cells were grown in DMEM with 10% FBS, 2 mM glutamine, and 1% penstrep and incubated at 37 °C in 5% CO_2_ in a humidified environment. All reagents were purchased from EuroClone (Milan, Italy). Briefly, the cell line was plated in 96 well plates at an adequate number to reach 80%–90% of confluence at the end of the assay. 16 h after cell plating, compounds were dissolved in DMSO to give a 10 mM stock solution, diluted in growth medium, and added at a final working concentration of 10 μM. After 48 h of incubation, 30 μL of 3‐(4,5‐dimethyl‐2‐thiazolyl)‐2,5‐diphenyl‐2H‐tetrazolium bromide (MTT) at a concentration of 2 mg mL^−1^ in PBS were added in each well. Then, after further 4 h of incubation, the supernatant was removed, and 100 μL/well of DMSO were used to dissolve the formazan precipitate that could be found in vital cells. After 20 min, the results were read at 570 nm by means of a plate reader. The results were expressed as percentage of the control samples in which the cells were incubated with the same amount of DMSO but without compounds. The assays were repeated three times. In each set, every single compound was tested six times. Variation among duplicates was less than 10%.

##### Plasmodium Cultures and Compound Susceptibility Assay

Continuous in vitro *Plasmodium falciparum* cultures were carried out according to Trager and Jensen with slight modifications.^[^ ^38^ ^]^ The CQ‐susceptible strain D10 and the CQ‐resistant strain W2 were maintained at 5% hematocrit (human type A‐positive red blood cells) in RPMI 1640 (EuroClone, Celbio) medium with the addition of 1% AlbuMax (Invitrogen, Milan, Italy), 0.01% hypoxanthine, 20 mM HEPES (at pH 7.4), and 2 mM glutamine. All the cultures were maintained at 37 °C in a low‐oxygen atmosphere consisting of 1% O_2_, 5% CO_2_, and 94% N_2_. Compounds were dissolved in DMSO to a stock concentration of 10 mg/ml and then diluted with complete medium to achieve the desired concentrations (final DMSO concentration <1%, which is nontoxic to the parasite). Derivatives were placed in 96‐well flat‐bottomed microplates in duplicate, and seven 1:2 serial dilutions were made directly in the plate in a volume of 100 μL. Asynchronous cultures with parasitemia of 1%–1.5% (assessed through Giemsa stained blood smears) and 1% final hematocrit were aliquoted into the plates and incubated for 72 h at 37 °C in a final volume of 200 μL/well. Uninfected erythrocytes at 2% hematocrit were used as blank. The antimalarial CQ was tested against the parasite strains as a positive control of inhibition. Parasite growth was determined spectrophotometrically (OD_650_) by measuring the activity of the parasite lactate dehydrogenase (pLDH), according to a modified version of the method of Makler in control and drug‐treated cultures.^[^ ^39^ ^]^ The antimalarial activity was expressed as 50% inhibitory concentrations (IC_50_). The IC_50_ values were extrapolated from nonlinear regression analysis of the concentration–response curve, using the software Gen5 1.10 provided with the Synergy 4 (BioTek) reader. Each IC_50_ value was the mean of three independent experiments run in duplicates.

##### Antileishmanial Evaluation

The promastigote stage of *L. infantum* strain MHOM/TN/80/IPT1 (kindly provided by Dr M. Gramiccia, ISS, Roma) and *L. tropica* strain (MHOM/IT/2012/ISS3130) were cultured in RPMI 1640 medium (EuroClone) supplemented with 10% heat‐inactivated fetal calf serum (EuroClone), 20 mM Hepes, and 2 mM L‐glutamine at 24 °C. To determine the 50% inhibitory concentration (IC_50_), the MTT method was used.^[^ ^40^, ^41^ ^]^ Compounds were dissolved in DMSO and then diluted with medium to achieve the required concentrations. Drugs were placed in 96 wells round‐bottom microplates and seven serial dilutions made. Amphotericin B was used as reference antiLeishmania drug. Parasites were diluted in complete medium to 5 × 10^6^ parasites mL^−1^ and 100 μL of the suspension was added to each well. The plates were incubated at 24 °C for 72 h, after the incubation 20 μL of MTT solution (5 mg mL^−1^) was added to each well and incubated for additional 3 h. The plates were then centrifuged at 1000 × g for 8 min at room temperature and the supernatants were discarded. The resulting pellets were dissolved in 100 μL of lysing buffer consisting of 20% w/v of a solution of SDS (Sigma), 40% of DMF (Merck) in H_2_O. The absorbance was measured spectrophotometrically at a test wavelength of 550 nm and a reference wavelength of 650 nm. The results were expressed as IC_50_ which was the concentration of compound necessary to inhibit parasite growth by 50%; each IC_50_ value was the mean of separate experiments performed in duplicate.

## Conflict of Interest

The authors declare no conflict of interest.

## Author Contributions

**Matteo Lusardi**: investigation (lead); methodology (lead); writing—original draft (lead). **Nicoletta Basilico**: investigation (lead); writing—review and editing (lead). **Erika Iervasi**: investigation (lead); writing—review and editing (lead). **Chiara Brullo**: methodology (lead); writing—review and editing (lead). **Silvia Parapini**: investigation (lead); methodology (lead). **Marco Ponassi**: investigation (lead); methodology (lead). **Camillo Rosano**: data curation (supporting). **Andrea Spallarossa**: conceptualization (lead); writing—original draft (lead).

## Supporting information

Supplementary Material

## Data Availability

The data that support the findings of this study are available from the corresponding author upon reasonable request.
