# Supplementary material for: Antiprotozoal Activity of Highly Substituted Pyrazole and Pyrimidine Derivatives
Source: ChemMedChem. 2025 Jun 29;20(16):e202500154. doi: 10.1002/cmdc.202500154 (PMC12368482; doi:10.1002/cmdc.202500154)
Supplement: Supplementary file 1 — Supplementary Material [file CMDC-20-e202500154-s001.pdf]

ChemMedChem

Supporting Information

Antiprotozoal activity of highly substituted pyrazole and pyrimidine derivatives

Lusardi Matteo,[a,b] Basilico Nicoletta,[c] Iervasi Erika,[d] Brullo Chiara,[a] Parapini Silvia,[e] Ponassi Marco,[d] Rosano Camillo,[d] Andrea Spallarossa[a,*]

[a] Department of Pharmacy, University of Genova, viale Benedetto XV, 3, 16132, Genova, Italy

[b] Molecular Modeling and Drug Discovery Laboratory, Istituto Italiano di Tecnologia, Via Morego, 30, 16163, Genova, Italy

[c] Dipartimento di Scienze Biomediche, Chirurgiche e Odontoiatriche, Università degli Studi di Milano, 20133 Milan, Italy

[d] Proteomics and Mass Spectrometry Unit, IRCCS Ospedale Policlinico San Martino, Largo R. Benzi 10, 16132, Genova, Italy

[e] Dipartimento di Scienze Biomediche per la Salute, Università degli Studi di Milano, 20133 Milan, Italy

Table of contents

Figure S1. ^{1}H-NMR (400 MHz, $CDCl_3$) spectrum of compound **12a (ML68)**
Figure S2. ^{1}H-NMR (400 MHz, $CDCl_3$) spectrum of compound **12b (ML65)**
Figure S3. ^{1}H-NMR (400 MHz, $CDCl_3$) spectrum of compound **12c (ML71)**
Figure S4. ^{1}H-NMR (400 MHz, DMSO-d_6) spectrum of compound **13a (ML99)**
Figure S5. ^{13}C-NMR (101 MHz, DMSO-d_6) spectrum of compound **13a (ML99)**
Figure S6. ^{1}H-NMR (400 MHz, DMSO-d_6) spectrum of compound **13b (ML97)**
Figure S7. ^{13}C-NMR (101 MHz, DMSO-d_6) spectrum of compound **13b (ML97)**
Figure S8. ^{1}H-NMR (400 MHz, $CDCl_3$) spectrum of compound **13c (M101)**
Figure S9. ^{13}C-NMR (101 MHz, $CDCl_3$) spectrum of compound **13c (ML101)**
Figure S10. ^{1}H-NMR (400 MHz, DMSO-d_6) spectrum of compound **14a (ML91)**
Figure S11. ^{13}C-NMR (101 MHz, DMSO-d_6) spectrum of compound **14a (ML91)**
Figure S12. ^{1}H-NMR (400 MHz, DMSO-d_6) spectrum of compound **14b (ML95)**
Figure S13. ^{13}C-NMR (101 MHz, DMSO-d_6) spectrum of compound **14b (ML95)**
Figure S14. ^{1}H-NMR (400 MHz, DMSO-d_6) spectrum of compound **14c (ML90)**
Figure S15. ^{13}C-NMR (101 MHz, DMSO-d_6) spectrum of compound **14c (ML90)**
Figure S16. ^{1}H-NMR (400 MHz, DMSO-d_6) spectrum of compound **14d (ML172)**
Figure S17. ^{13}C-NMR (101 MHz, DMSO-d_6) spectrum of compound **14d (ML172)**
Figure S18. ^{1}H-NMR (400 MHz, DMSO-d_6) spectrum of compound **14e (ML170)**
Figure S19. ^{13}C-NMR (101 MHz, DMSO-d_6) spectrum of compound **14e (ML170)**
Figure S20. ^{1}H-NMR (400 MHz, DMSO-d_6) spectrum of compound **14f (ML183)**
Figure S21. ^{13}C-NMR (101 MHz, DMSO-d_6) spectrum of compound **14f (ML183)**
Figure S22. ^{1}H-NMR (400 MHz, DMSO-d_6) spectrum of compound **15a (ML70)**
Figure S23. ^{13}C-NMR (101 MHz, DMSO-d_6) spectrum of compound **15a (ML70)**
Figure S24. ^{1}H-NMR (400 MHz, DMSO-d_6) spectrum of compound **15b (ML72)**
Figure S25. ^{13}C-NMR (101 MHz, DMSO-d_6) spectrum of compound **15b (ML72)**
Figure S26. ^{1}H-NMR (400 MHz, DMSO-d_6) spectrum of compound **15c (ML73**
Figure S27. ^{13}C-NMR (101 MHz, DMSO-d_6) spectrum of compound **15c (ML73)**
Figure S28. ^{1}H-NMR (400 MHz, DMSO-d_6) spectrum of compound **16a (ML203/2)**
Figure S29. ^{13}C-NMR (101 MHz, DMSO-d_6) spectrum of compound **16a (ML203/2)**
Figure S30. ^{1}H-NMR (400 MHz, DMSO-d_6) spectrum of compound **16b (ML209)**
Figure S31. ^{13}C-NMR (101 MHz, DMSO-d_6) spectrum of compound **16b (ML209)**
Figure S32. ^{1}H-NMR (400 MHz, DMSO-d_6) spectrum of compound **16e (ML206)**
Figure S33. ^{13}C-NMR (101 MHz, DMSO-d_6) spectrum of compound **16e (ML206)**
Figure S34. ^{1}H-NMR (400 MHz, DMSO-d_6) spectrum of compound **17a (ML210)**
Figure S35. ^{13}C-NMR (101 MHz, DMSO-d_6) spectrum of compound **17a (ML210)**
Figure S36. ^{1}H-NMR (400 MHz, DMSO-d_6) spectrum of compound **17b (ML217)**
Figure S37. ^{13}C-NMR (101 MHz, DMSO-d_6) spectrum of compound **17b (ML217)**
Figure S38. ^{1}H-NMR (400 MHz, DMSO-d_6) spectrum of compound **17e (ML214)**
Figure S39. ^{13}C-NMR (101 MHz, DMSO-d_6) spectrum of compound **17e (ML214)**
Table S1. Predicted Absorption and distribution properties of compounds **13-17**
Table S2. Predicted metabolism and excretion properties of compounds **13-17**
Table S3. Predicted toxicity profile of compounds **13-17**
Table S4. Predicted Toxicity pathways of compounds **13-17**

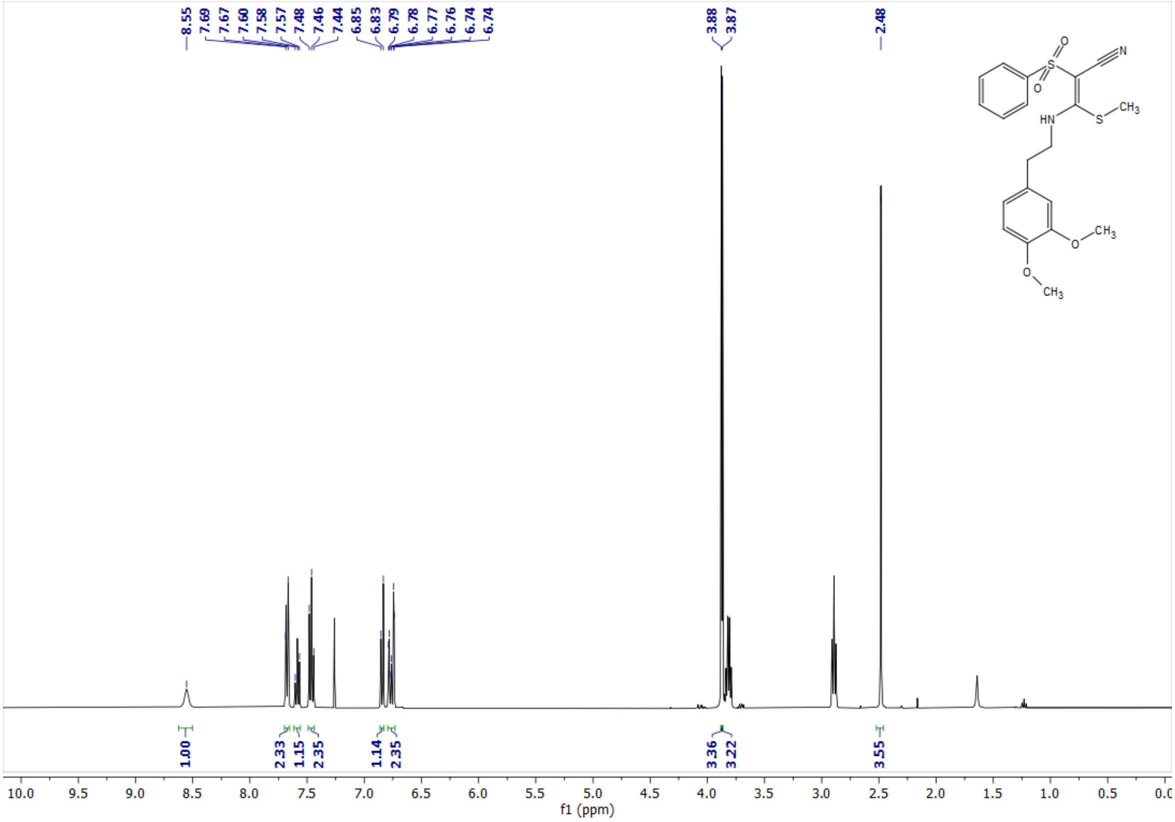

Figure S1. ^{1}H-NMR (400 MHz, $CDCl_3$) spectrum of compound **12a**

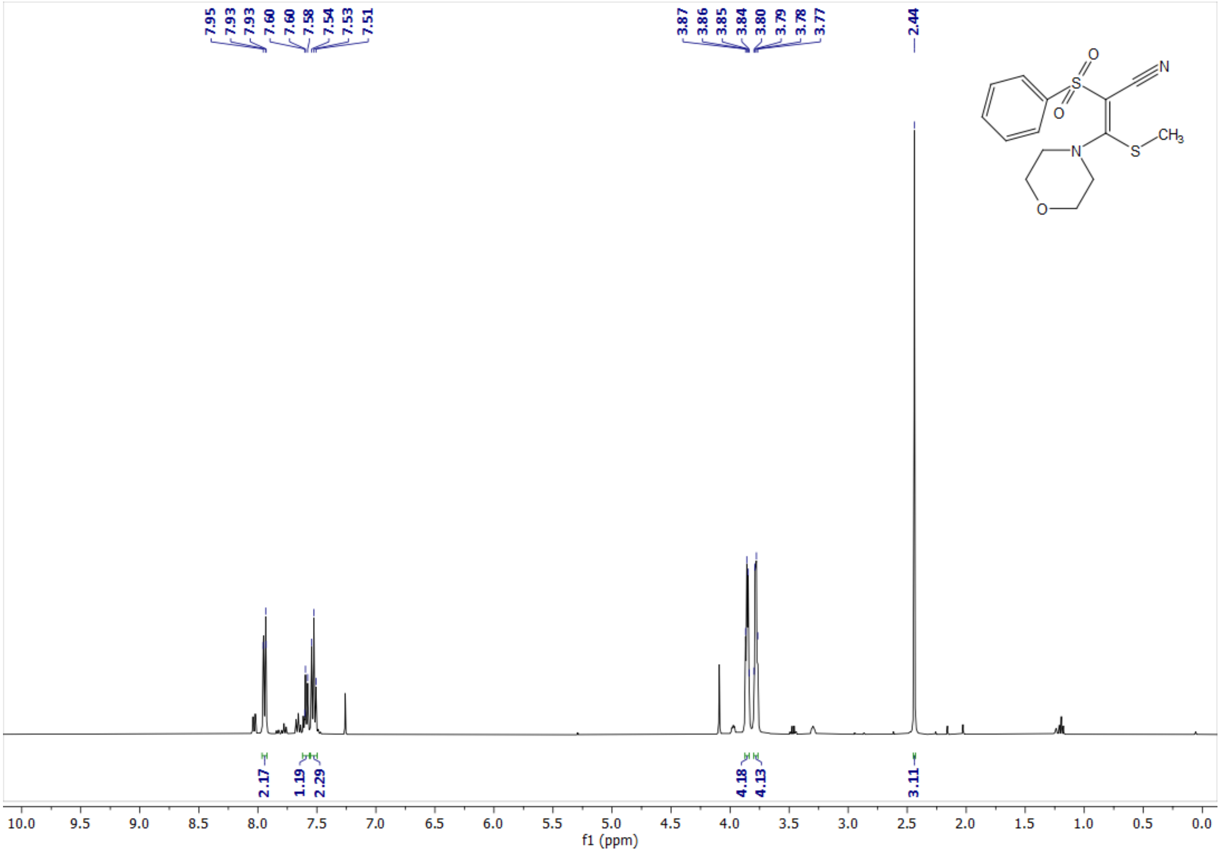

Figure S2. ^{1}H-NMR (400 MHz, $CDCl_3$) spectrum of compound **12b**

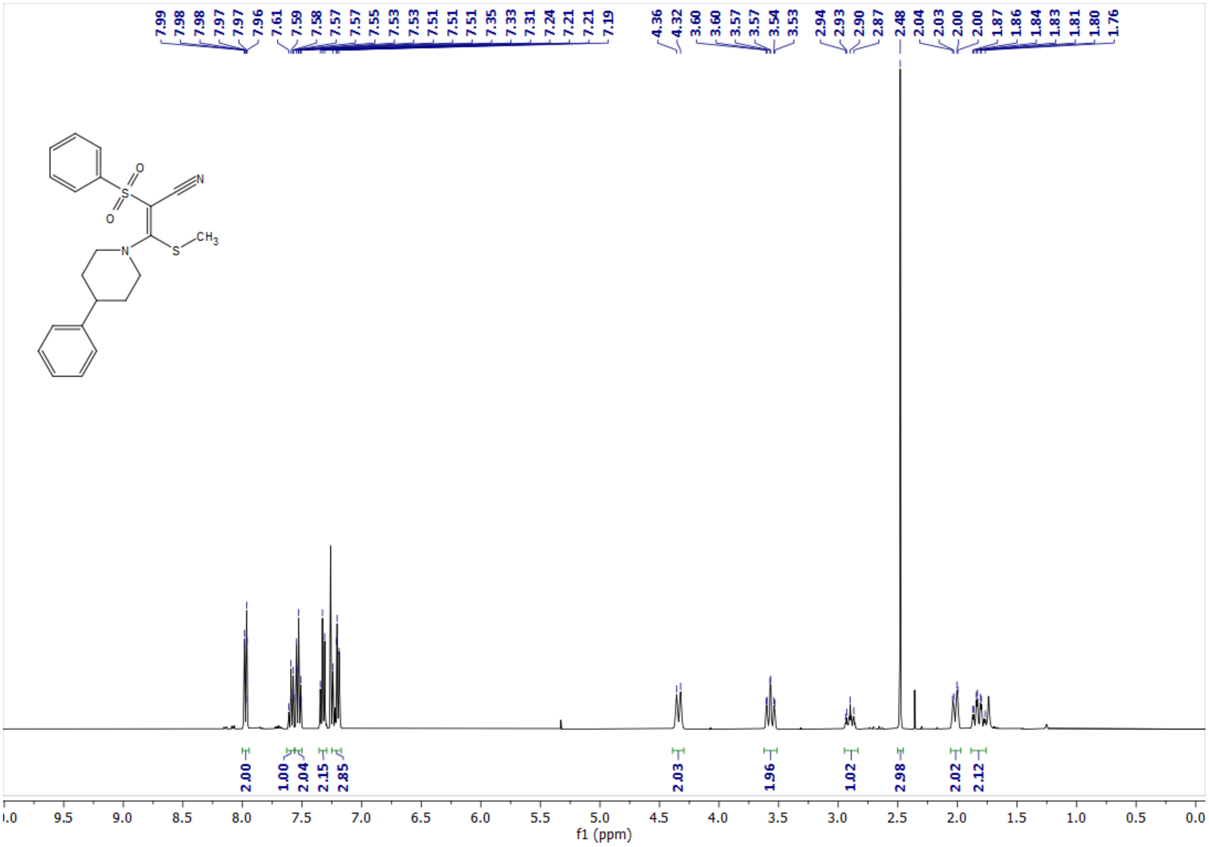

Figure S3. ^{1}H-NMR (400 MHz, $CDCl_3$) spectrum of compound **12c**

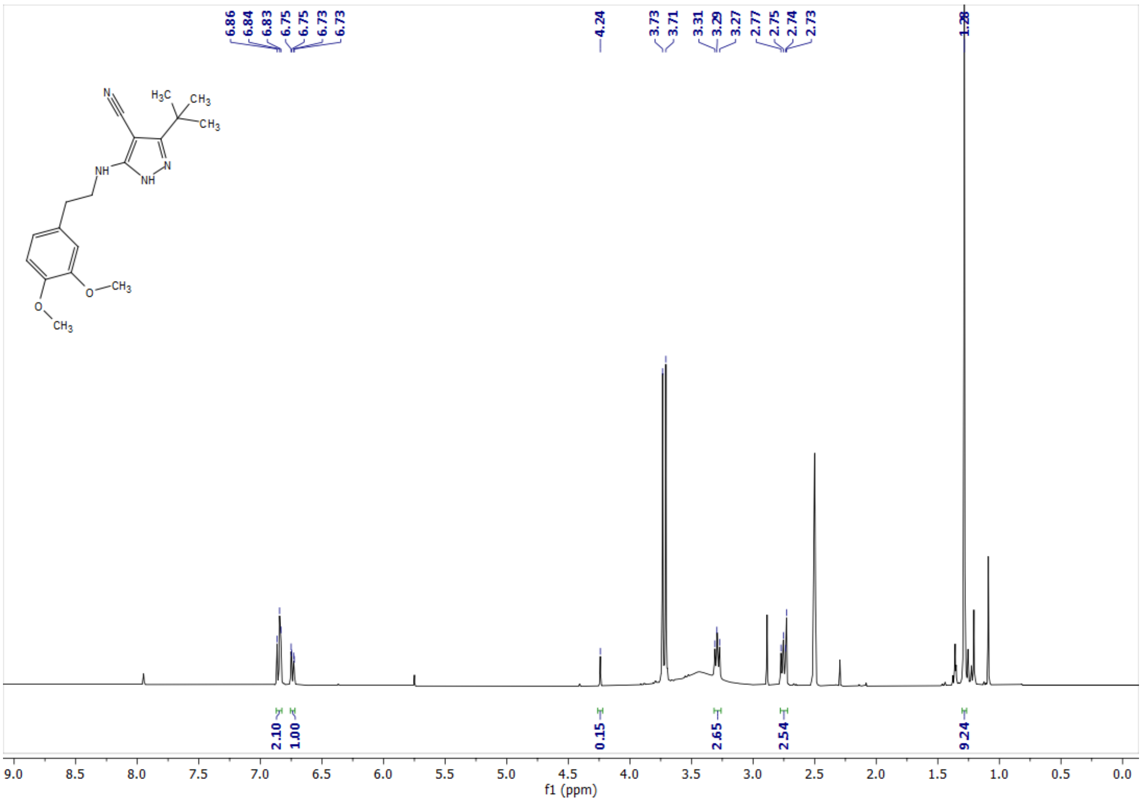

Figure S4. ^{1}H-NMR (400 MHz, DMSO-d_6) spectrum of compound **13a**

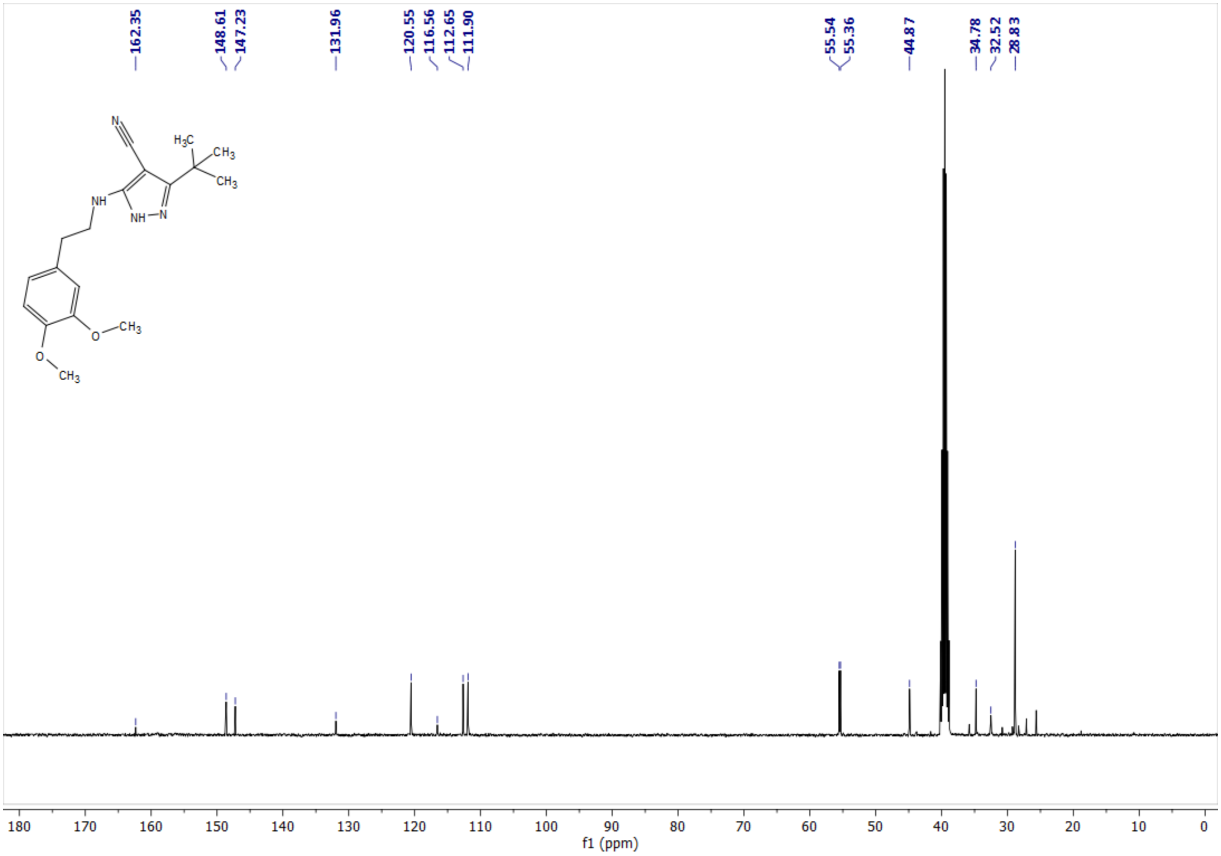

Figure S5. ^{13}C-NMR (101 MHz, DMSO-d$_6$) spectrum of compound **13a**

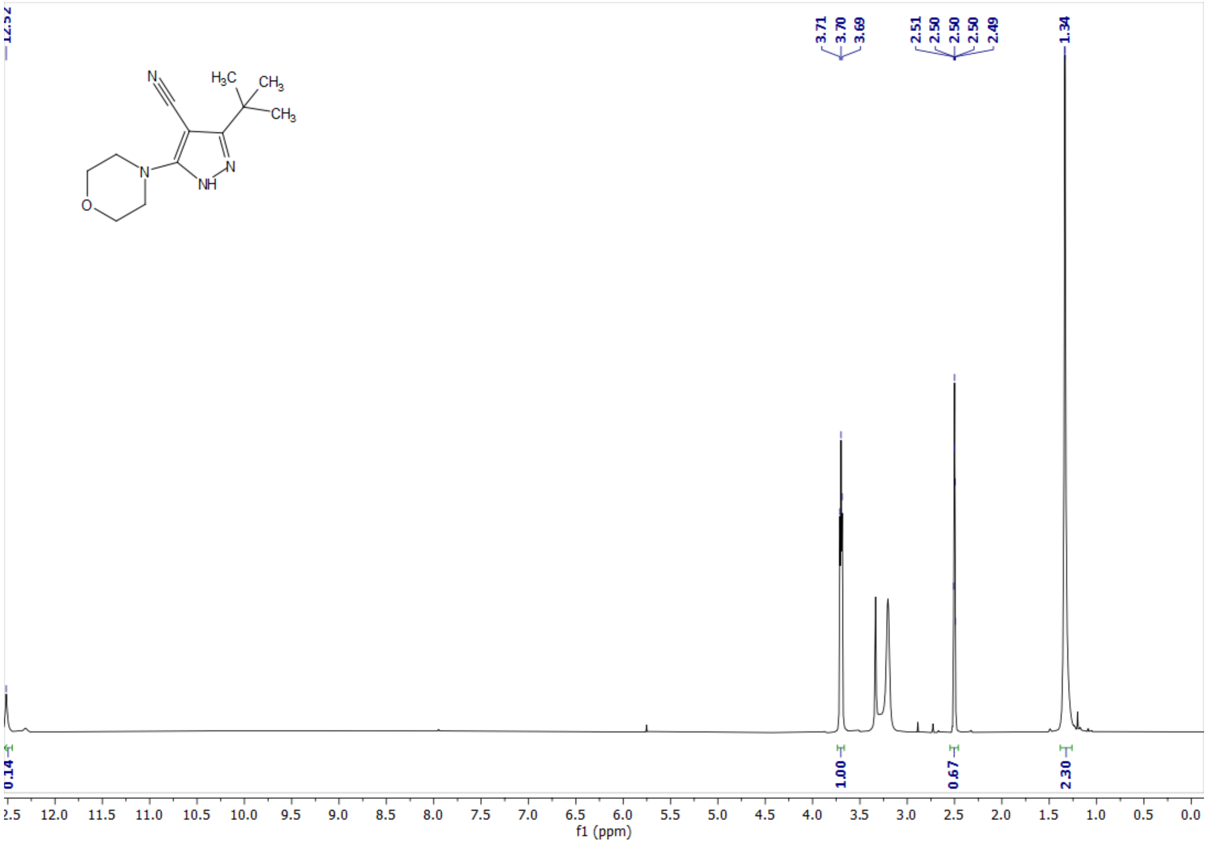

Figure S6. ^{1}H-NMR (400 MHz, DMSO-d$_6$) spectrum of compound **13b**

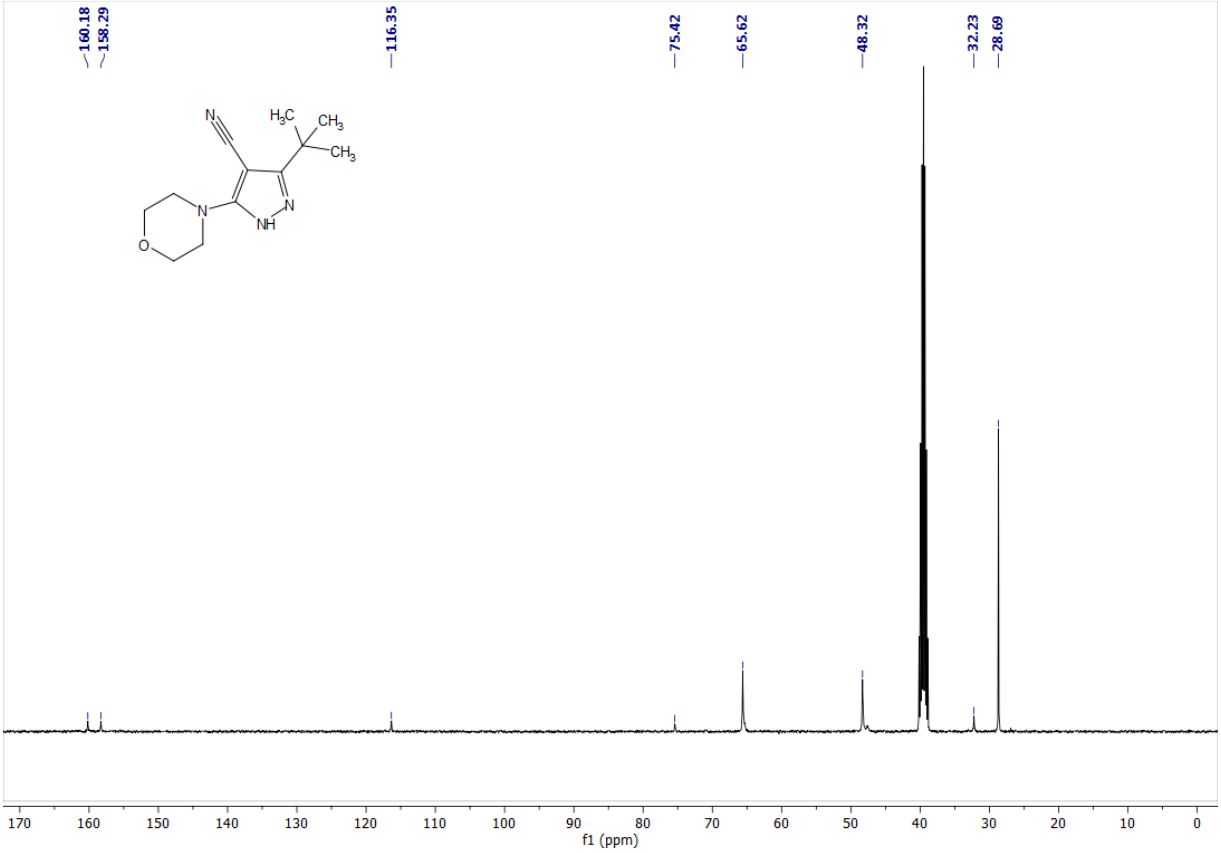

Figure S7. ^{13}C-NMR (101 MHz, DMSO-d$_6$) spectrum of compound **13b**

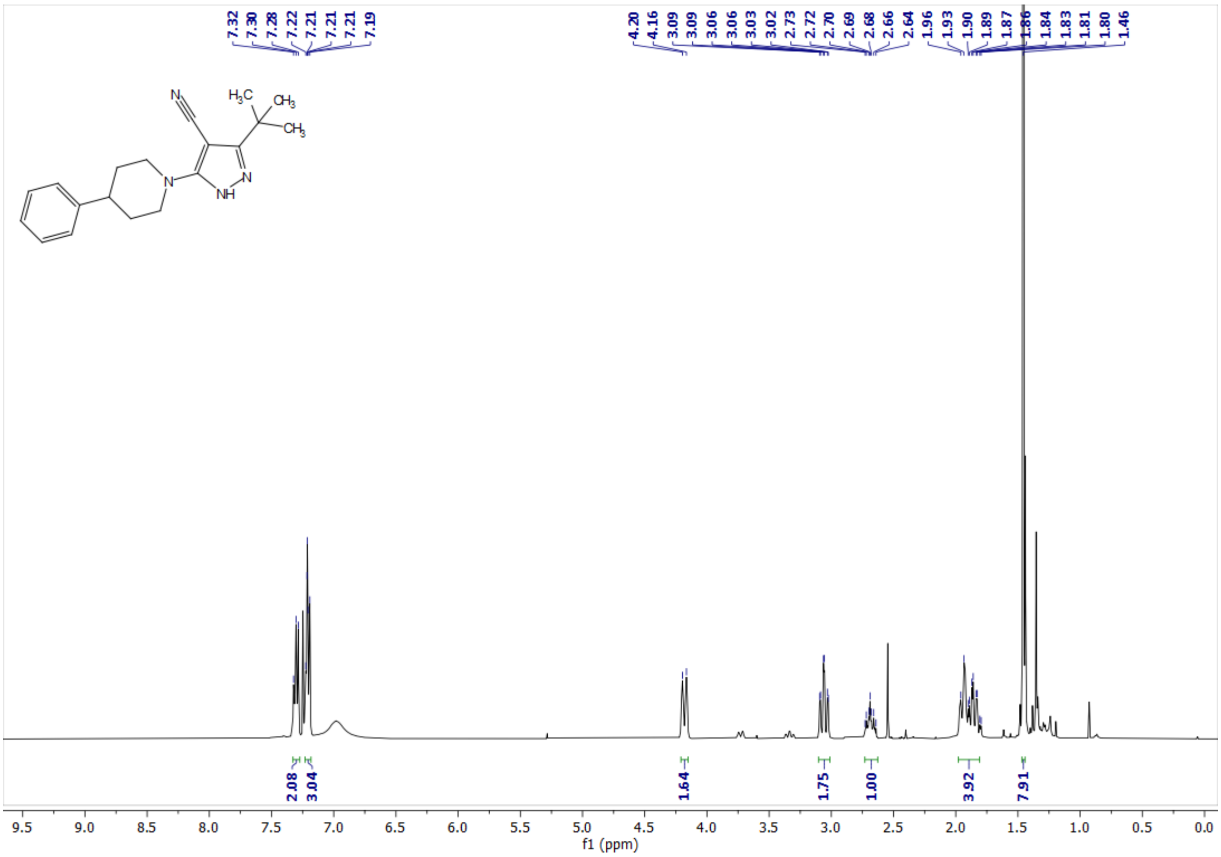

Figure S8. ^{1}H-NMR (400 MHz, CDCl$_3$) spectrum of compound **13c**

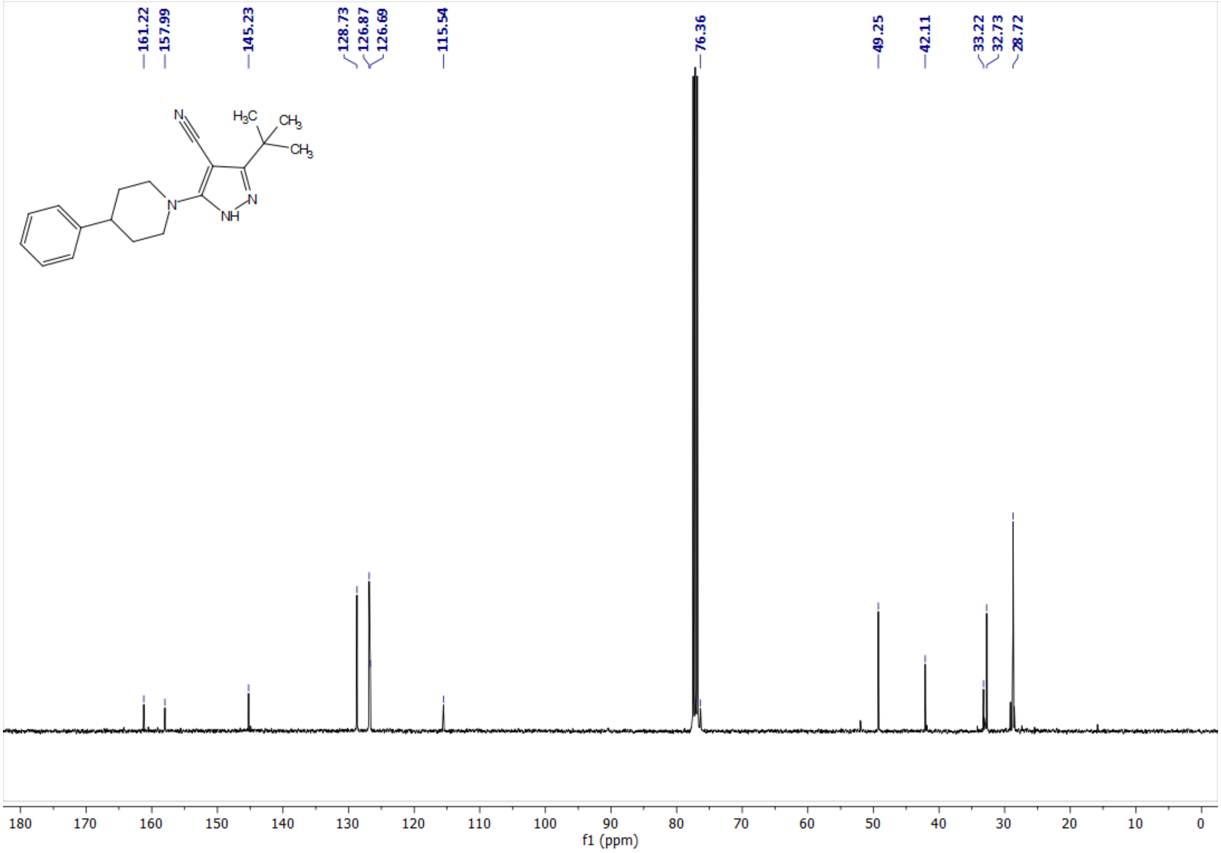

Figure S9. ^{13}C-NMR (101 MHz, $CDCl_3$) spectrum of compound **13c**

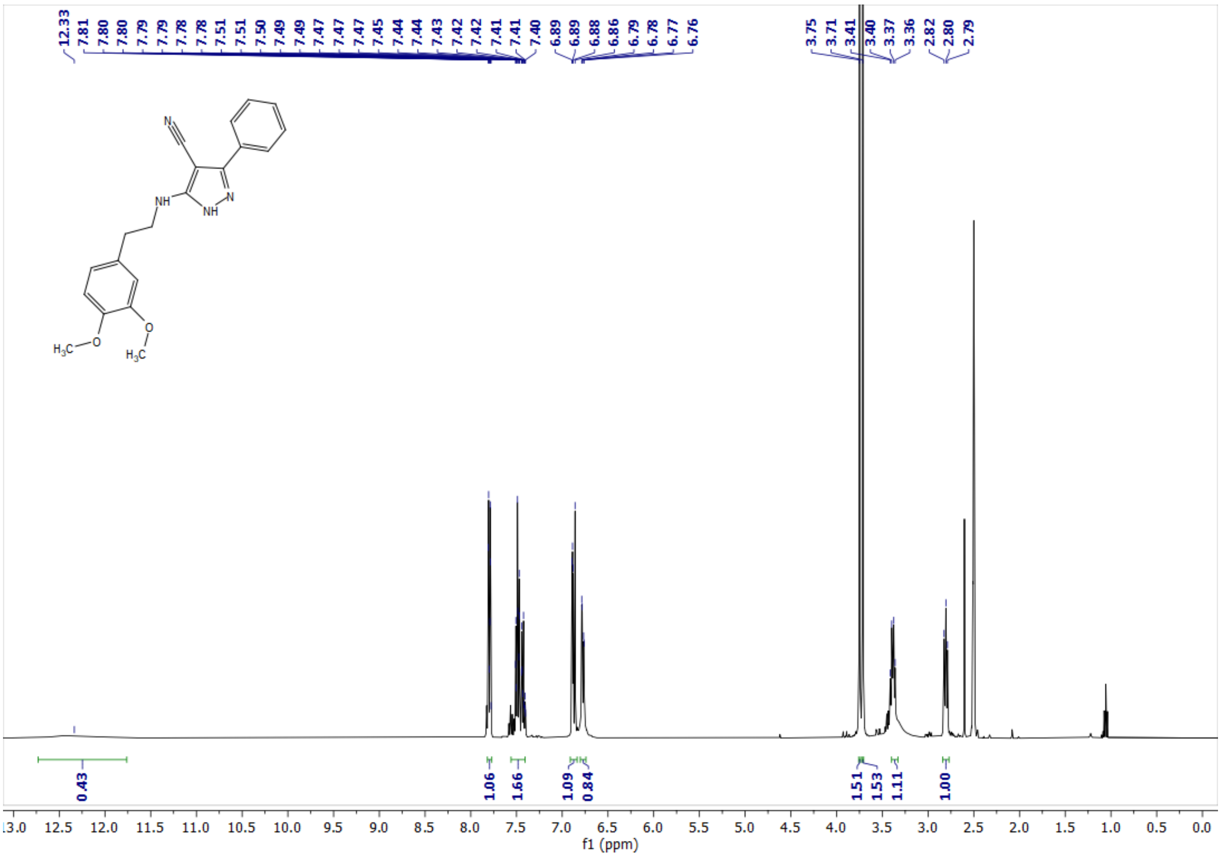

Figure S10. ^{1}H-NMR (400 MHz, DMSO-d_6) spectrum of compound **14a**

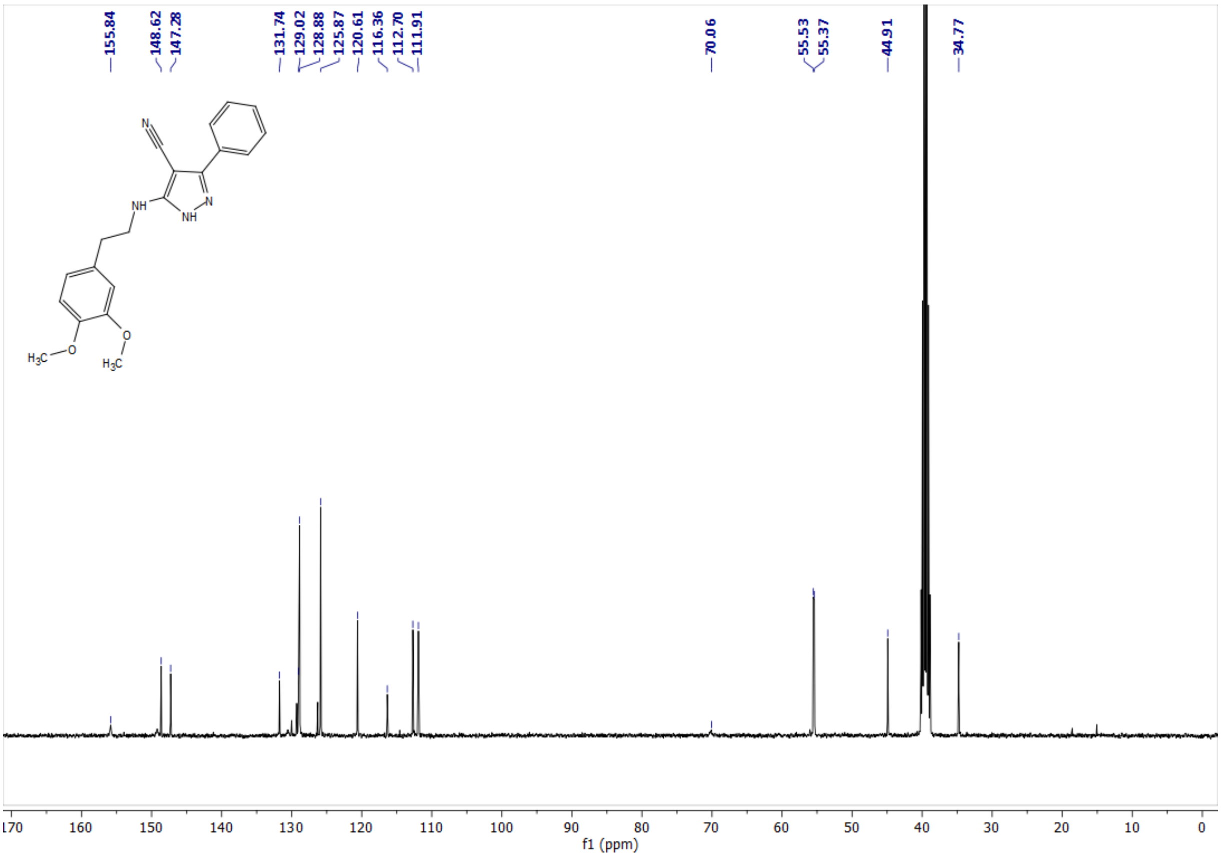

Figure S11. ^{13}C-NMR (101 MHz, DMSO-d$_6$) spectrum of compound **14a**

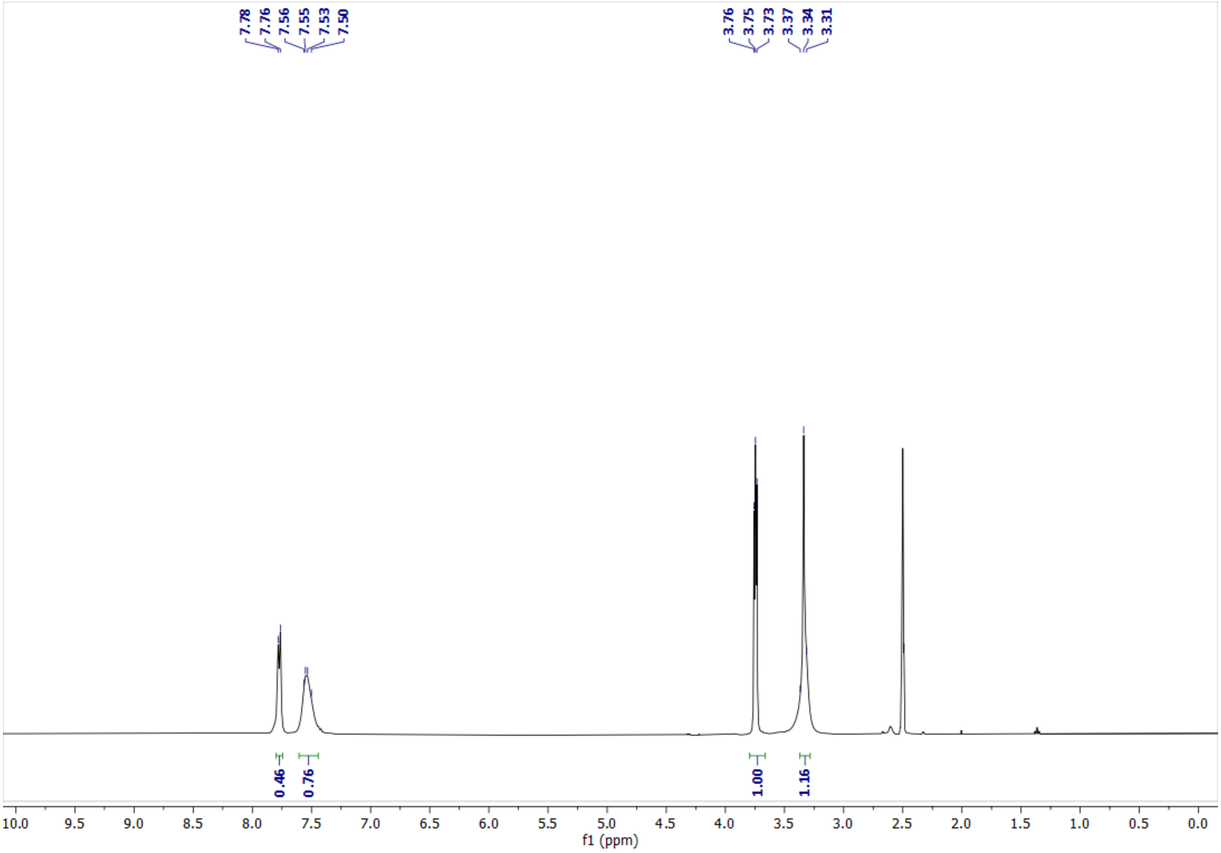

Figure S12. ^{1}H-NMR (400 MHz, DMSO-d$_6$) spectrum of compound 14**b**

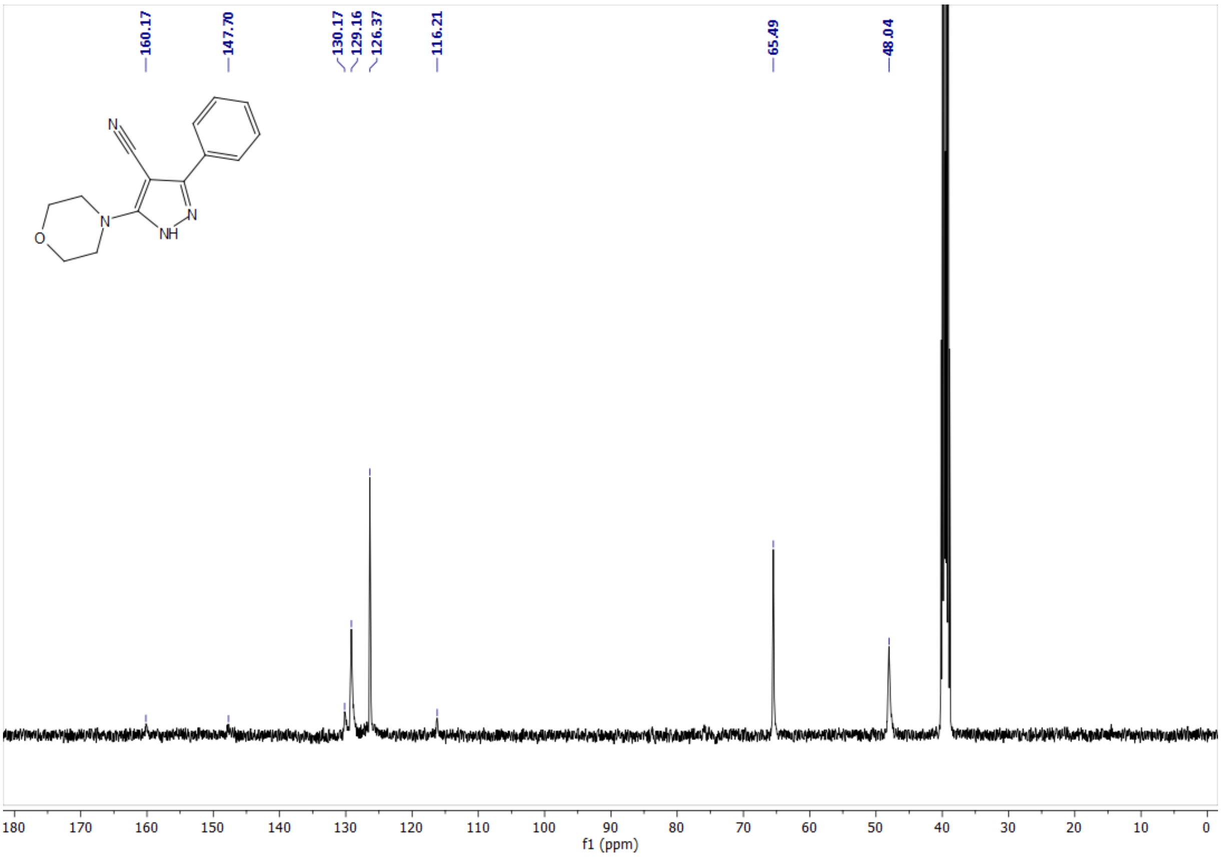

Figure S13. ^{13}C-NMR (101 MHz, DMSO-d$_6$) spectrum of compound **14b**

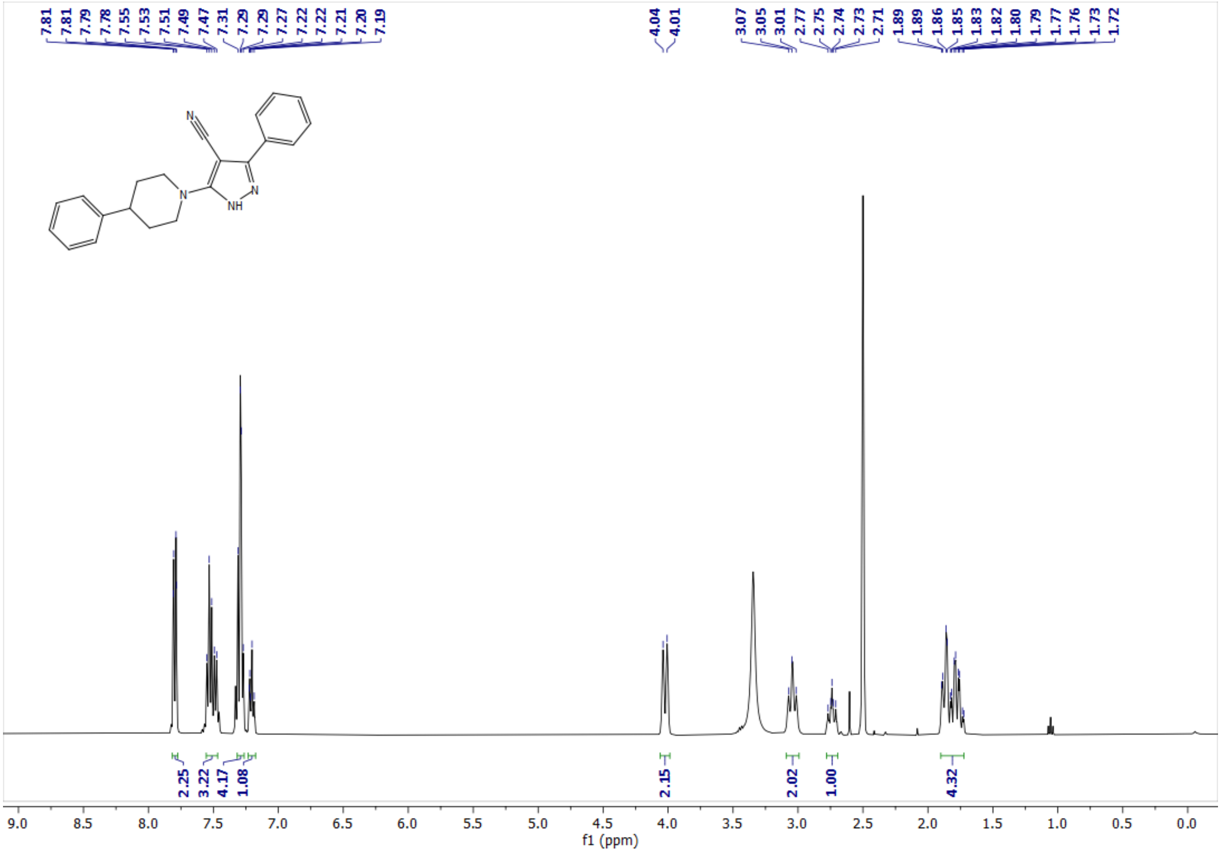

Figure S14. ^{1}H-NMR (400 MHz, DMSO-d$_6$) spectrum of compound **14c**

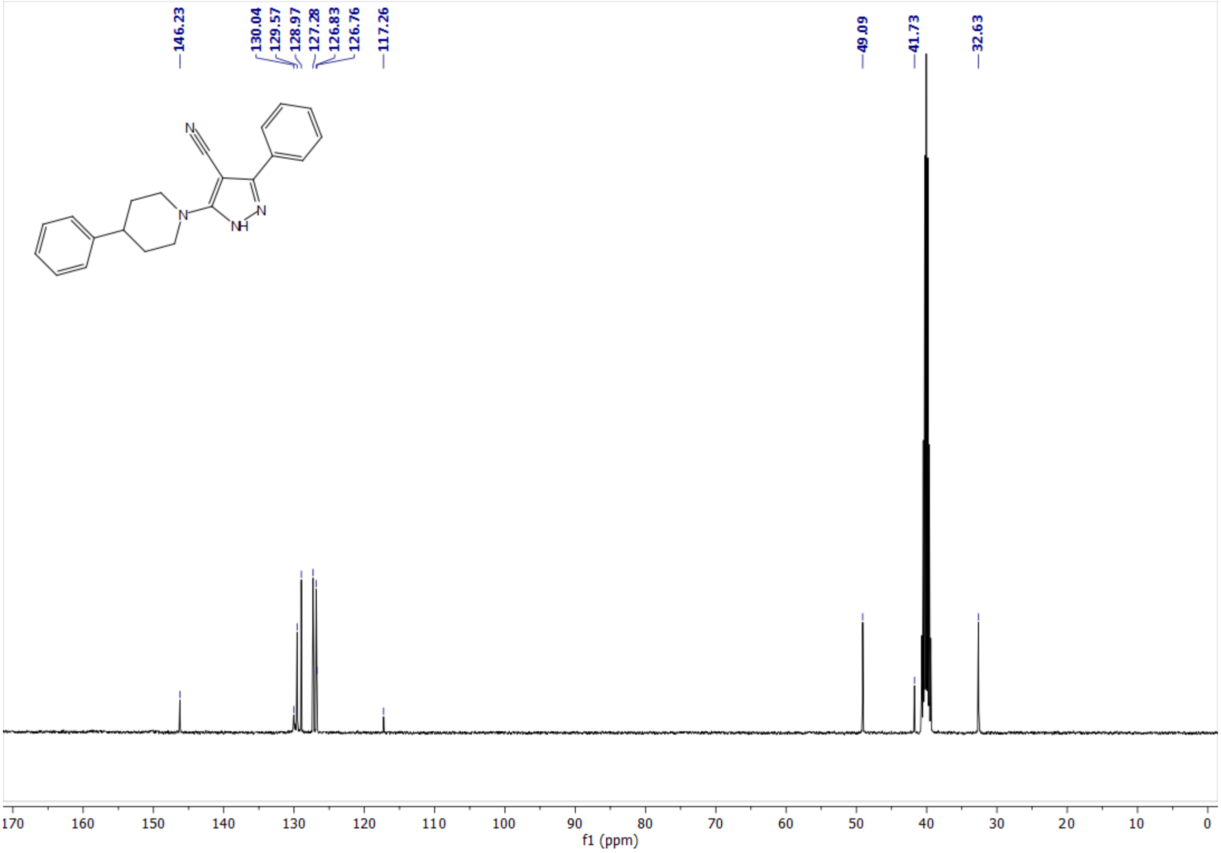

Figure S15. ^{13}C-NMR (101 MHz, DMSO-d$_6$) spectrum of compound **14c**

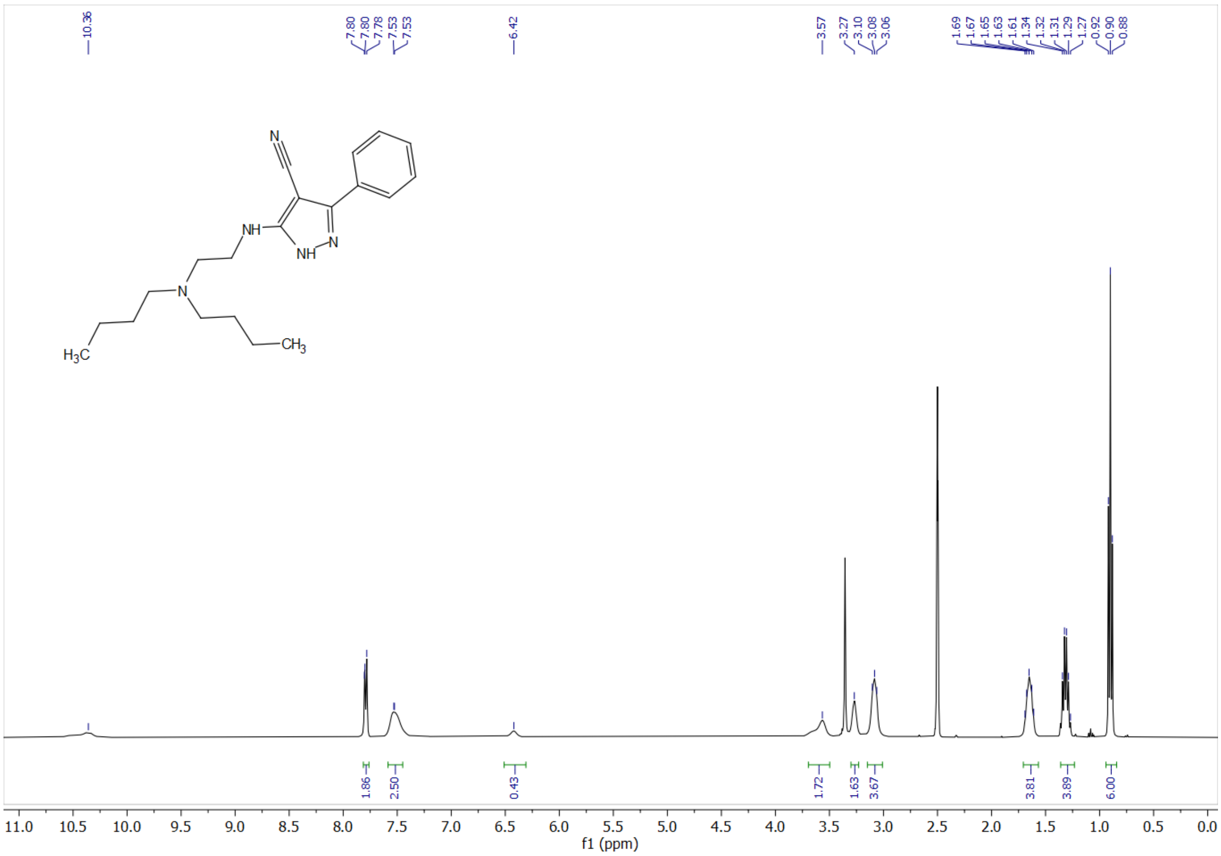

Figure S16. ^{1}H-NMR (400 MHz, DMSO-d$_6$) spectrum of compound **14d**

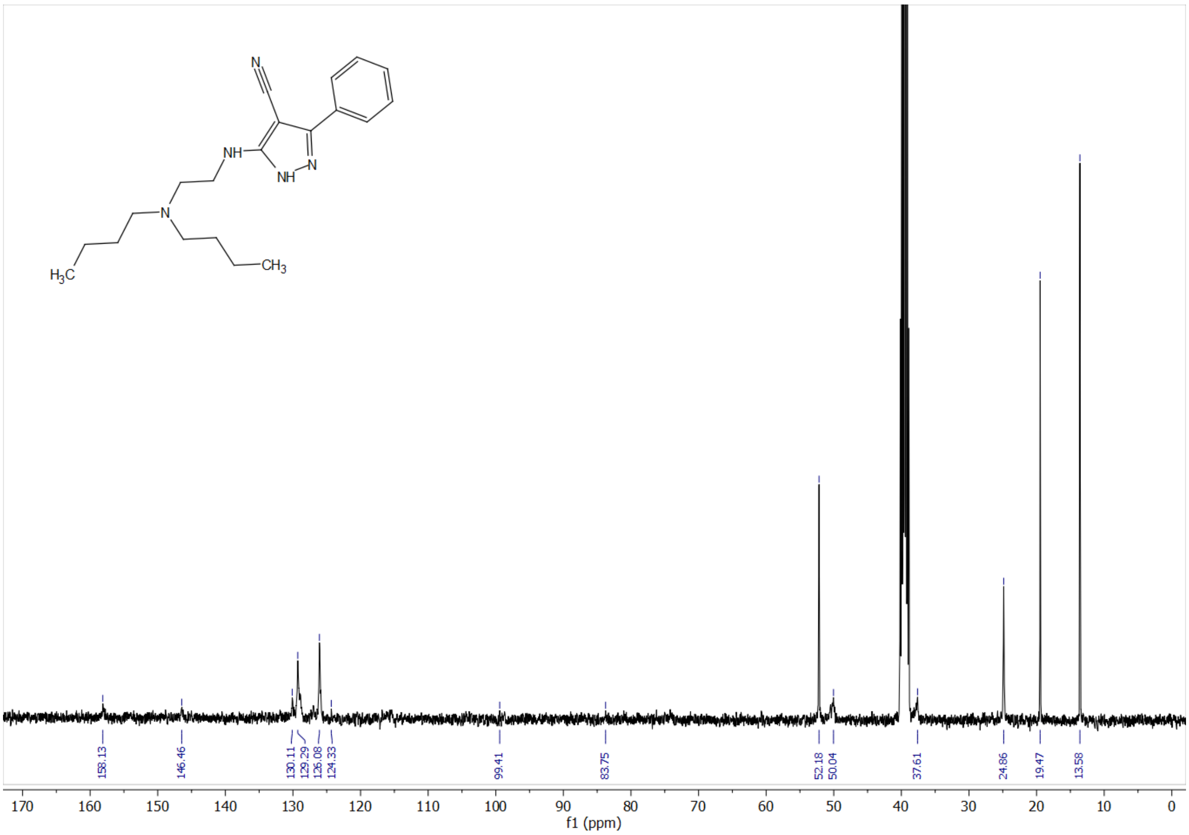

Figure S17. ^{13}C-NMR (101 MHz, DMSO-d$_6$) spectrum of compound **14d**

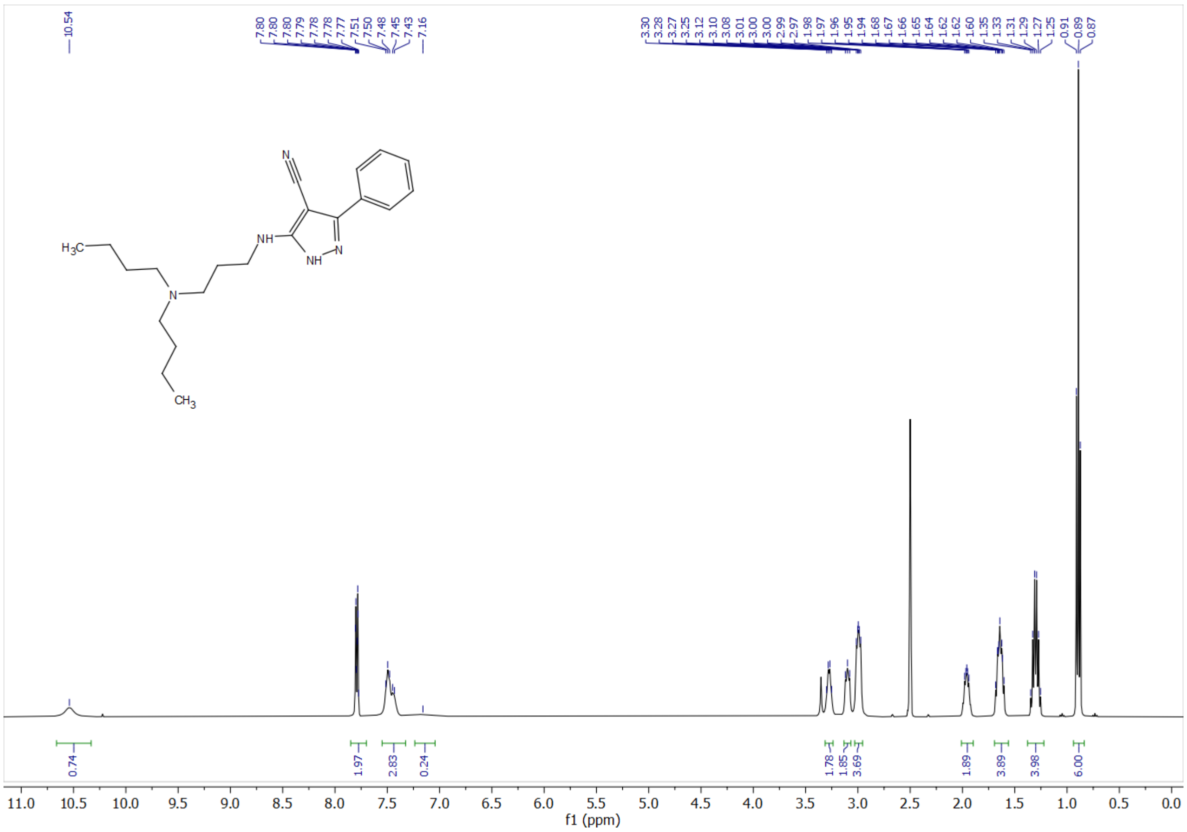

Figure S18. ^{1}H-NMR (400 MHz, DMSO-d$_6$) spectrum of compound **14e**

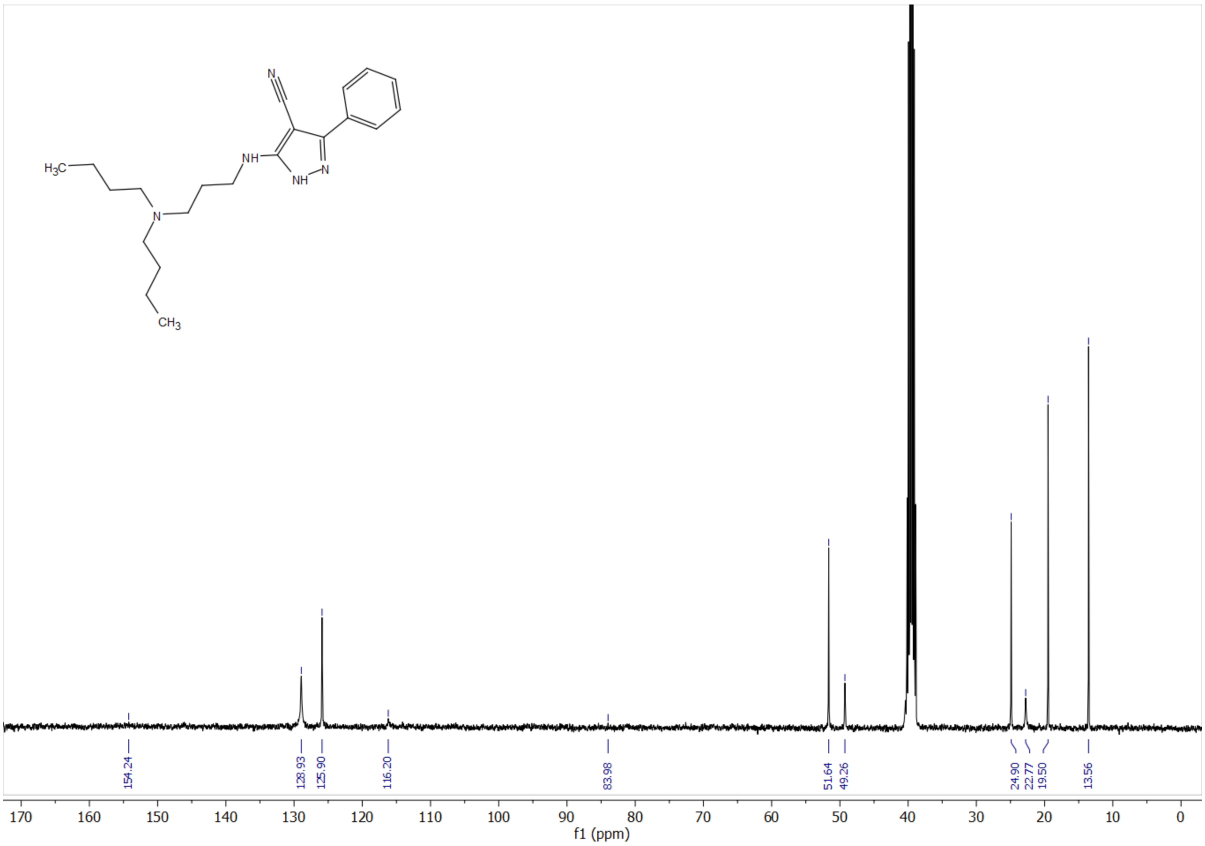

Figure S19. ^{13}C-NMR (101 MHz, DMSO-d_6) spectrum of compound **14e**

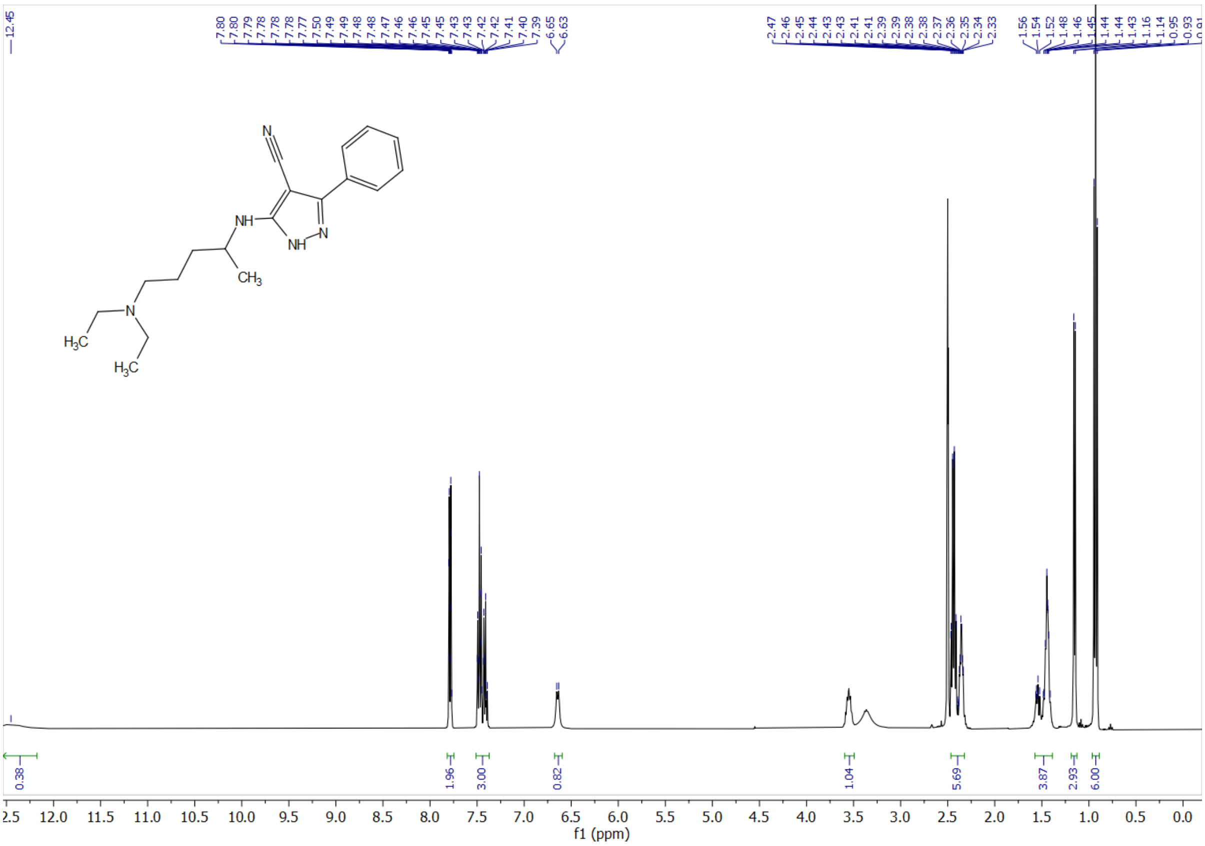

Figure S20. ^{1}H-NMR (400 MHz, DMSO-d_6) spectrum of compound **14f**

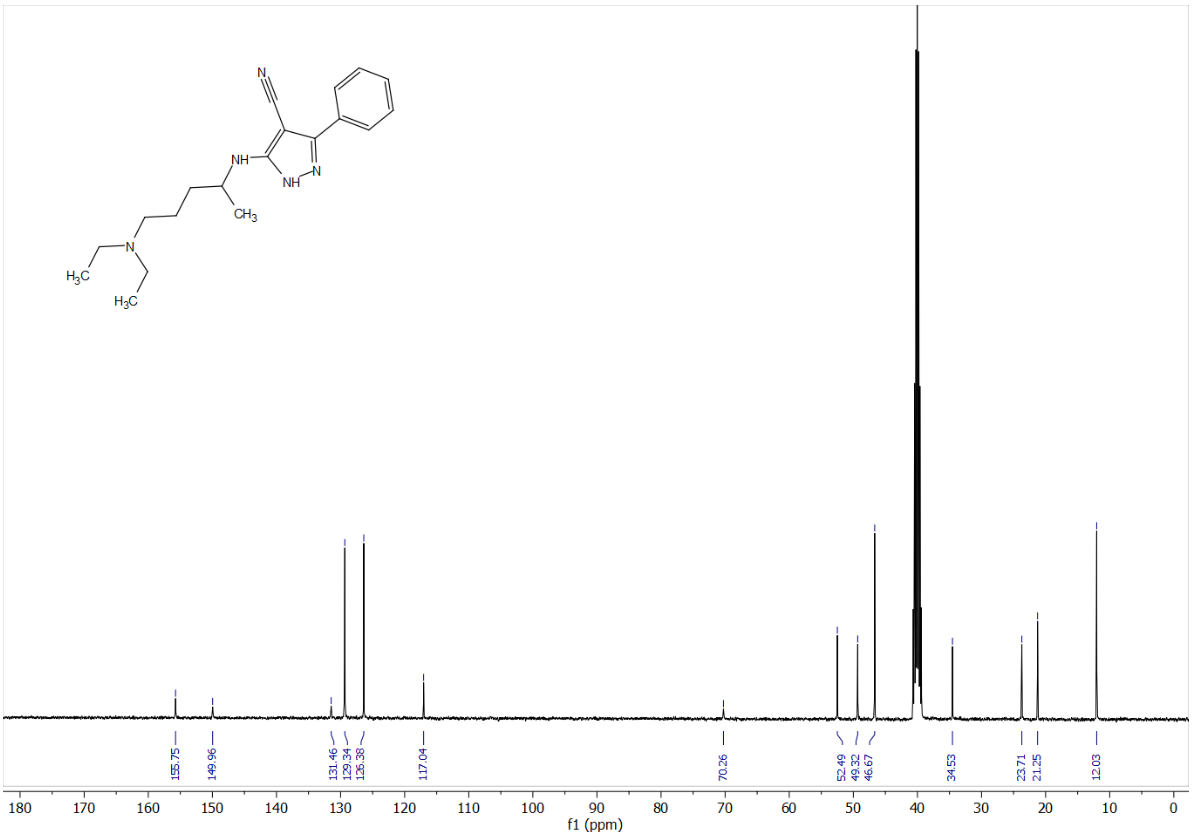

Figure S21. ^{13}C-NMR (101 MHz, DMSO-d$_6$) spectrum of compound **14f**

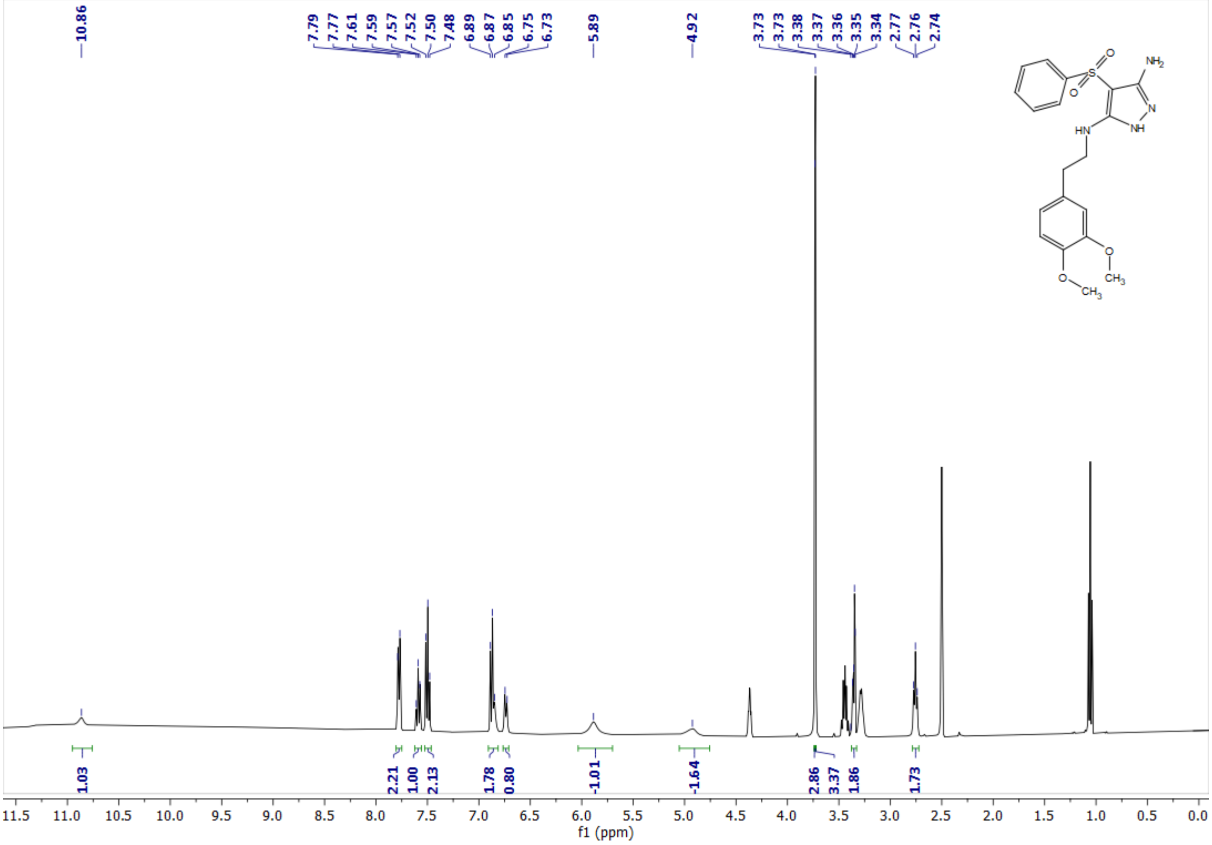

Figure S22. ^{1}H-NMR (400 MHz, DMSO-d$_6$) spectrum of compound **15a**

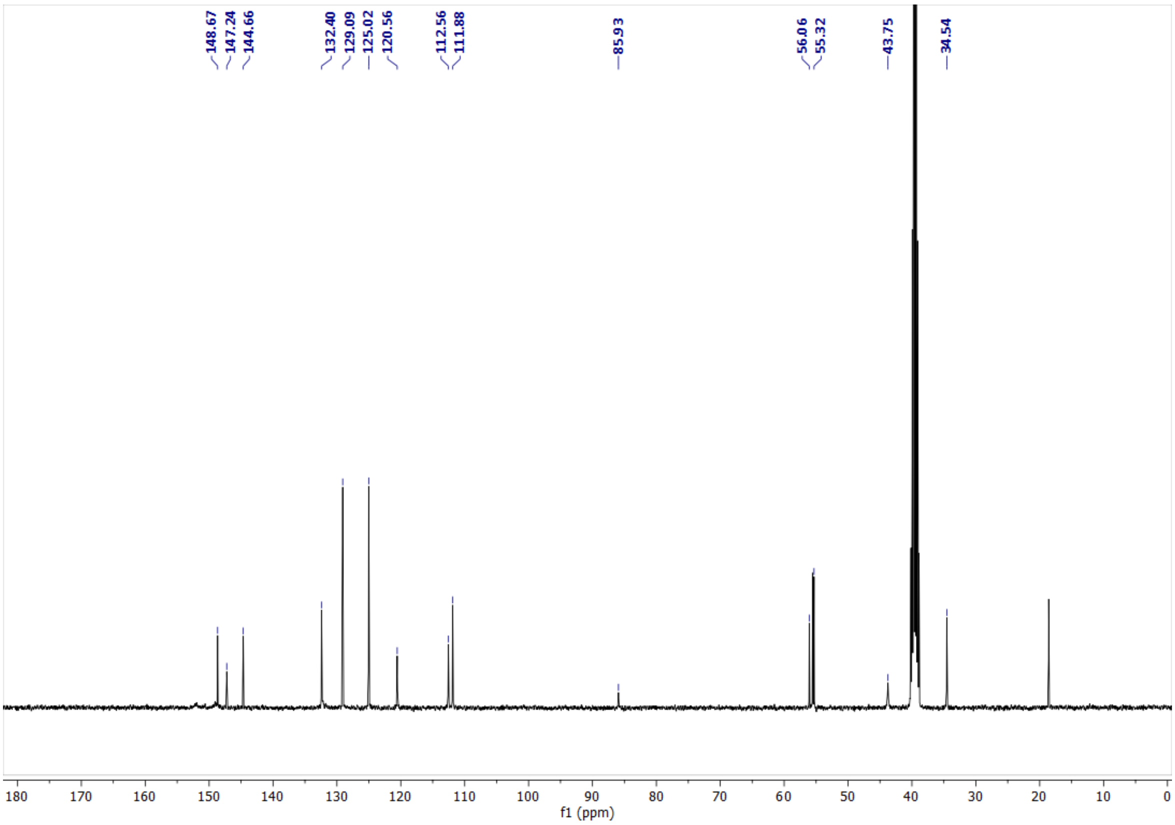

Figure S23. ^{13}C-NMR (101 MHz, DMSO-d_6) spectrum of compound **15a**

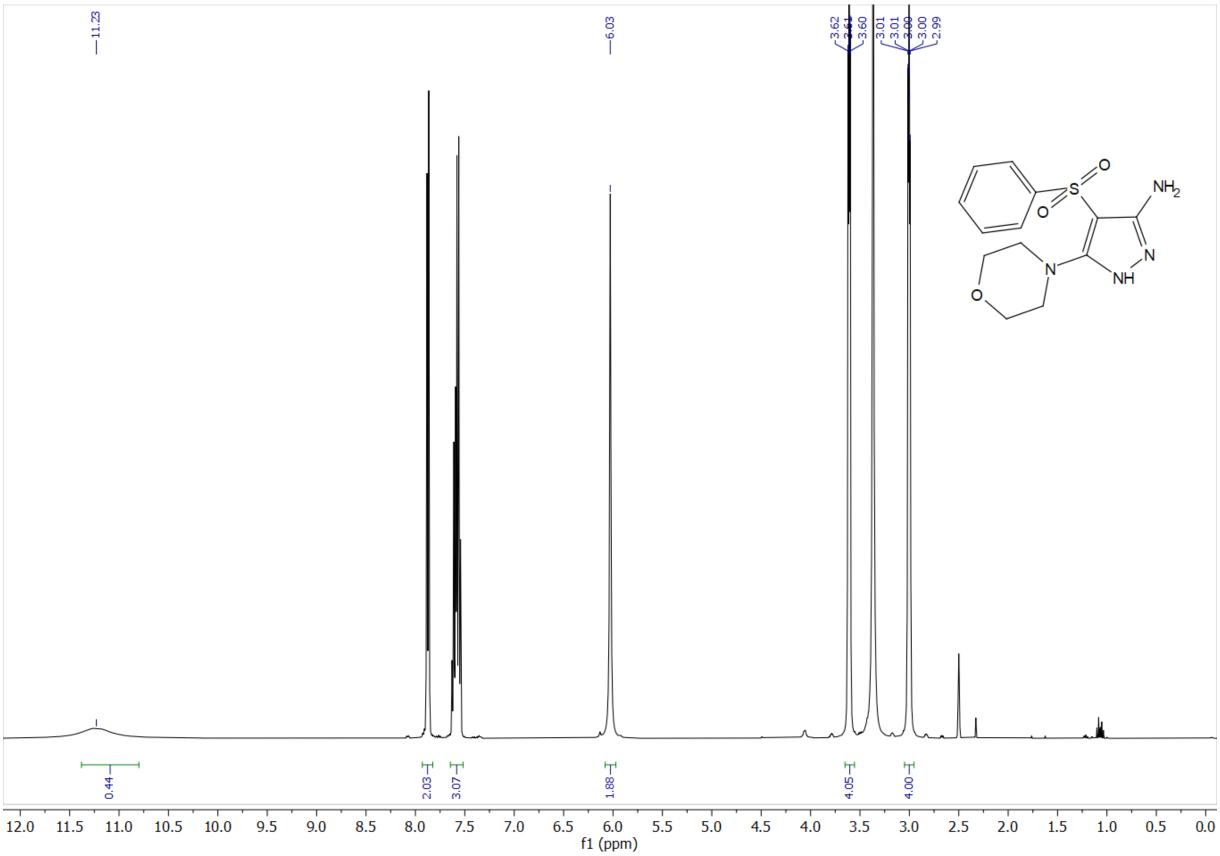

Figure S24. ^{1}H-NMR (400 MHz, DMSO-d_6) spectrum of compound **15b**

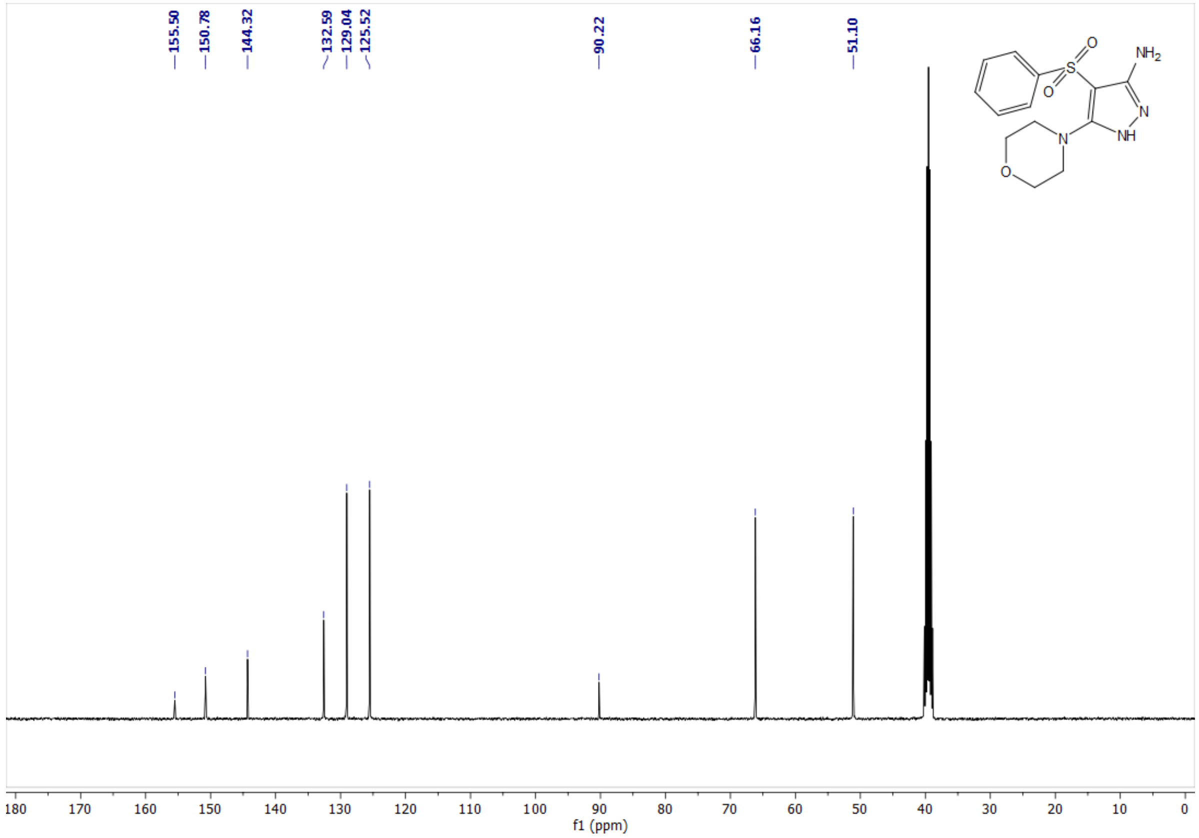

Figure S25. ^{13}C-NMR (101 MHz, DMSO-d$_6$) spectrum of compound **15b**

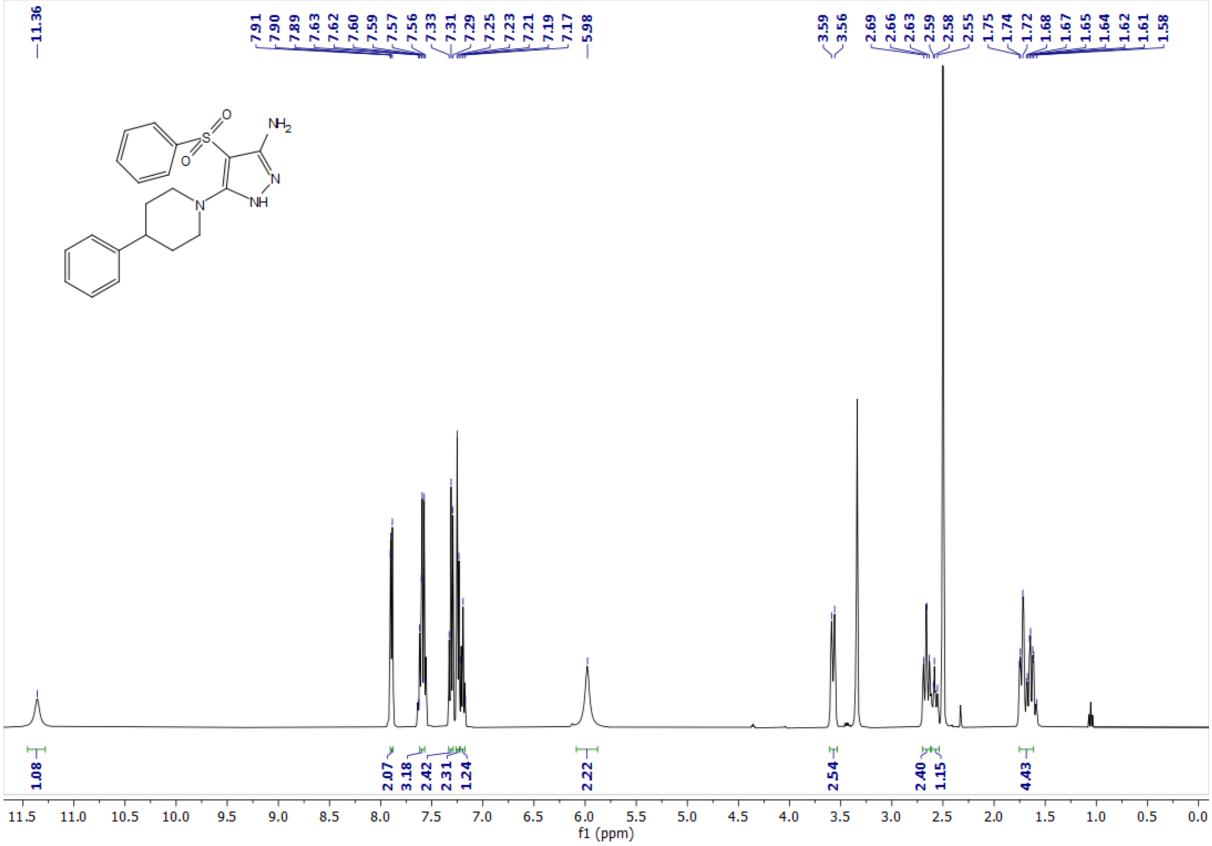

Figure S26. ^{1}H-NMR (400 MHz, DMSO-d$_6$) spectrum of compound **15c**

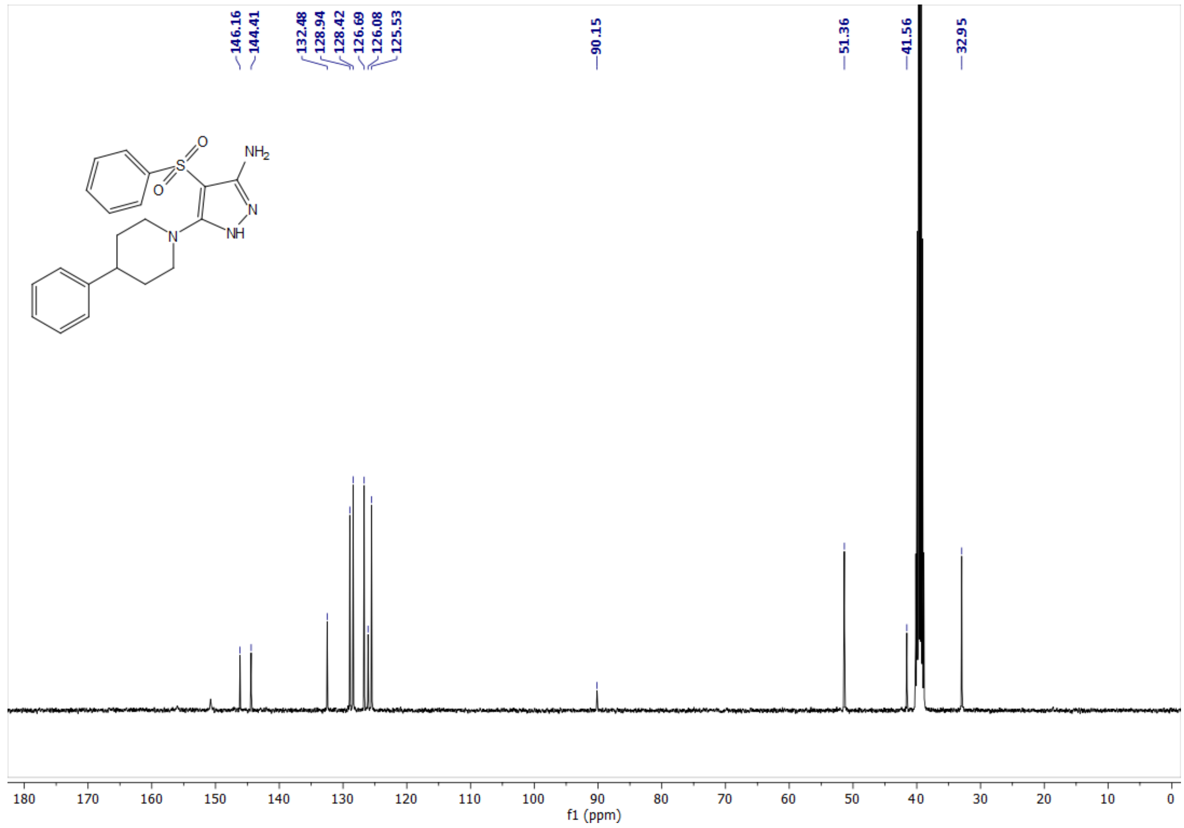

Figure S27. ^{13}C-NMR (101 MHz, DMSO-d_6) spectrum of compound **15c**

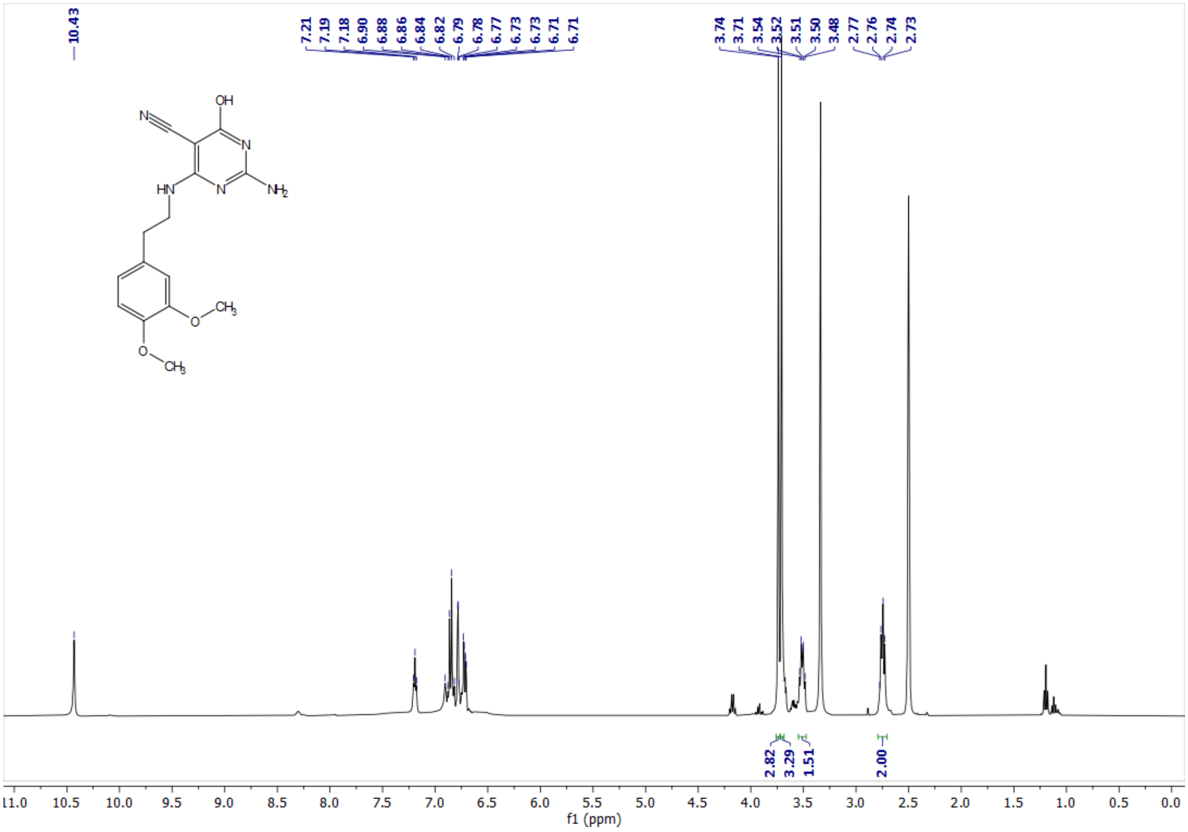

Figure S28. ^{1}H-NMR (400 MHz, DMSO-d_6) spectrum of compound **16a**

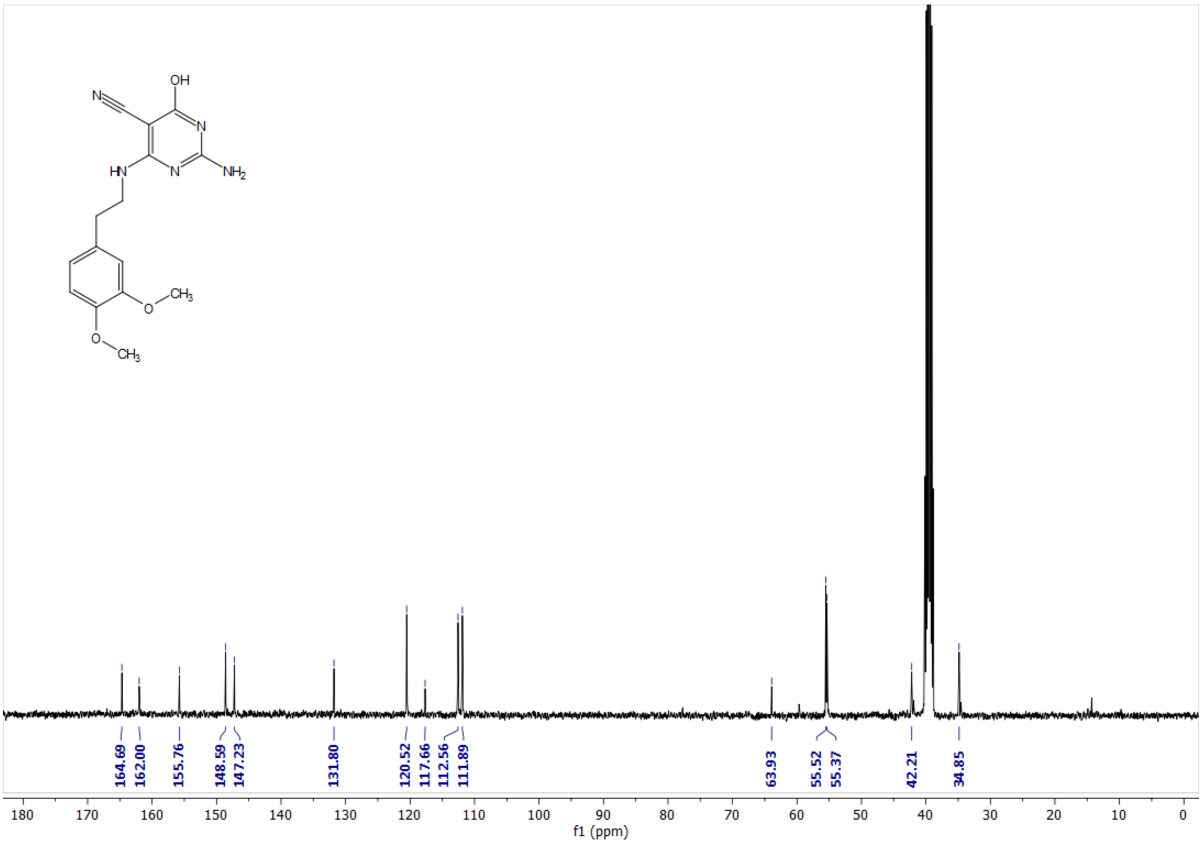

Figure S29. ^{13}C-NMR (101 MHz, DMSO-d_6) spectrum of compound **16a**

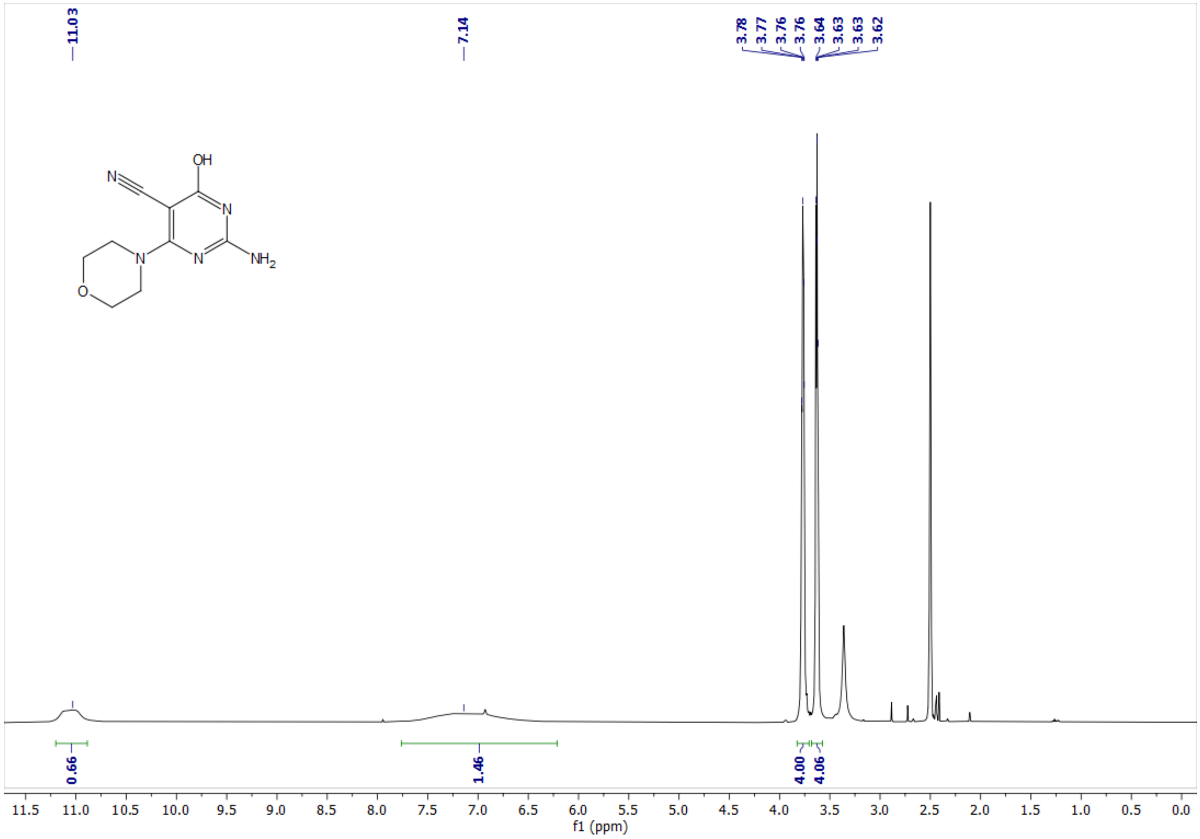

Figure S30. ^{1}H-NMR (400 MHz, DMSO-d_6) spectrum of compound **16b**

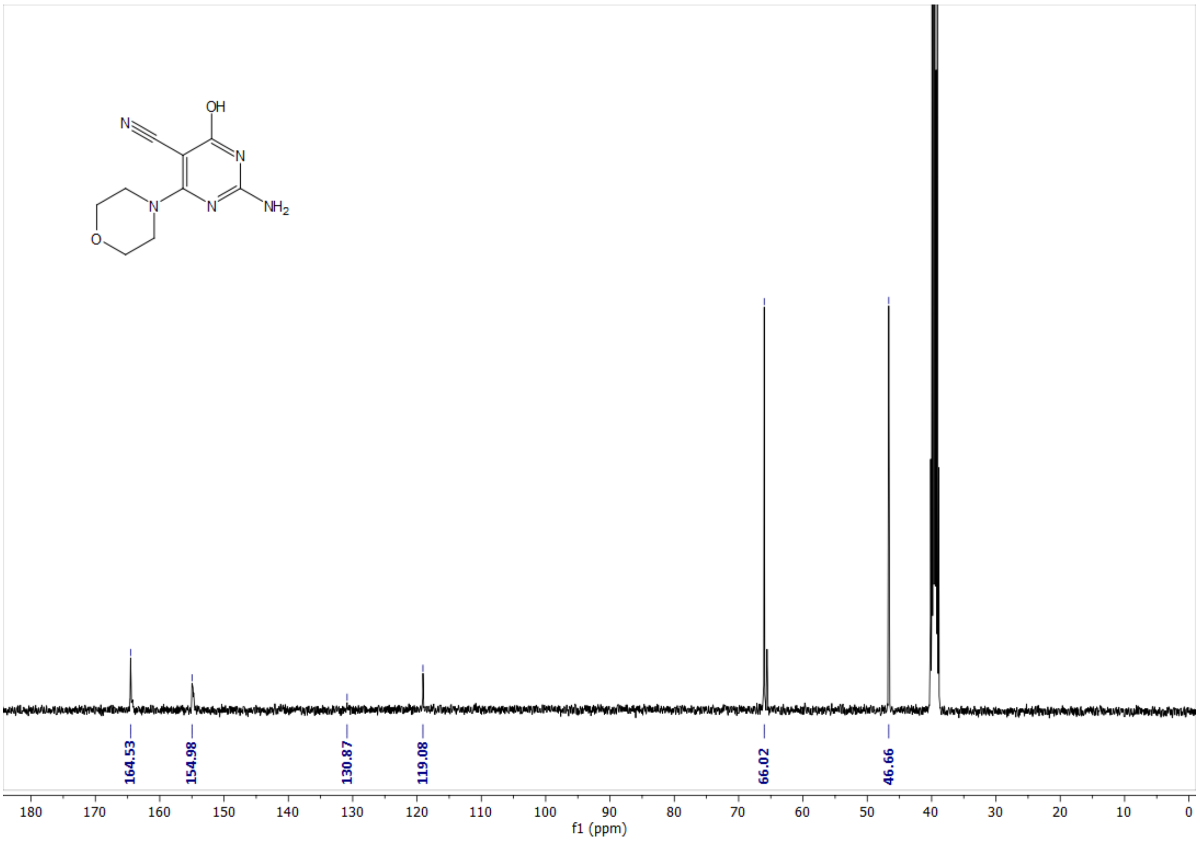

Figure S31. ^{13}C-NMR (101 MHz, DMSO-d$_6$) spectrum of compound **16b**

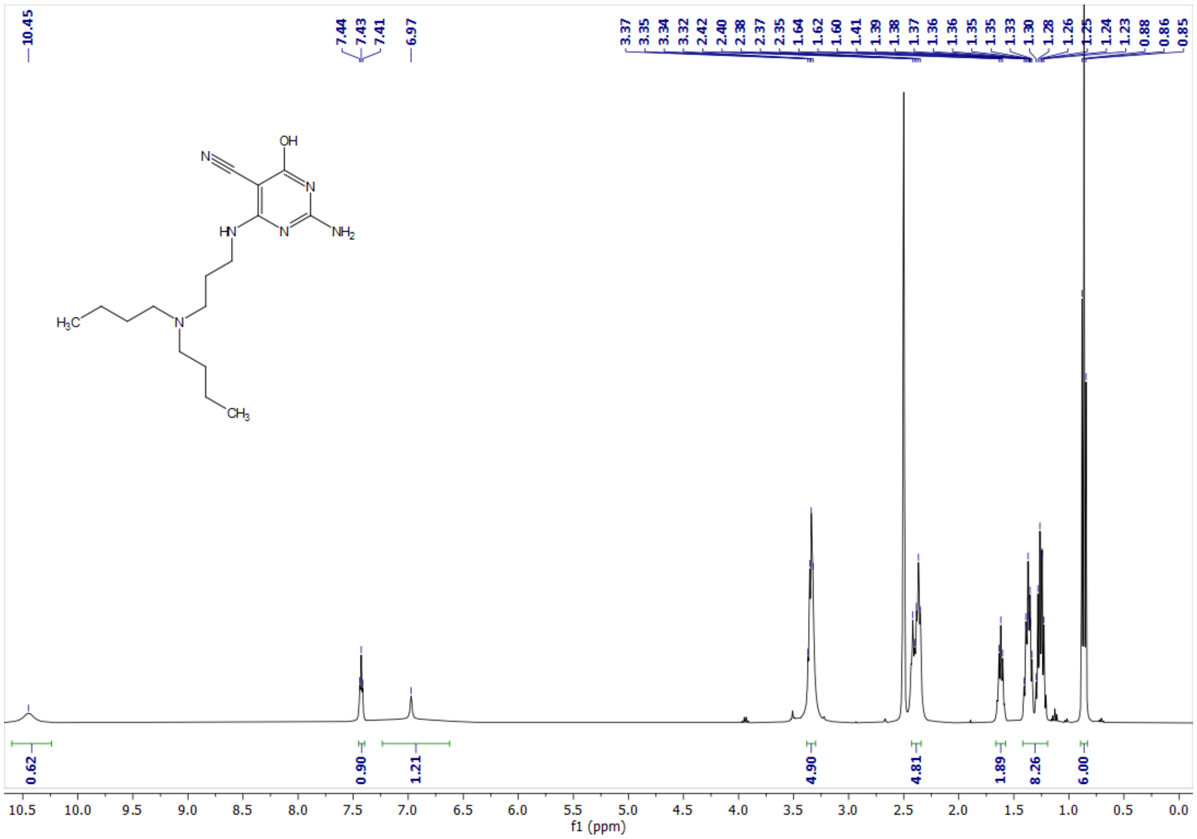

Figure S32. ^{1}H-NMR (400 MHz, DMSO-d$_6$) spectrum of compound **16e**

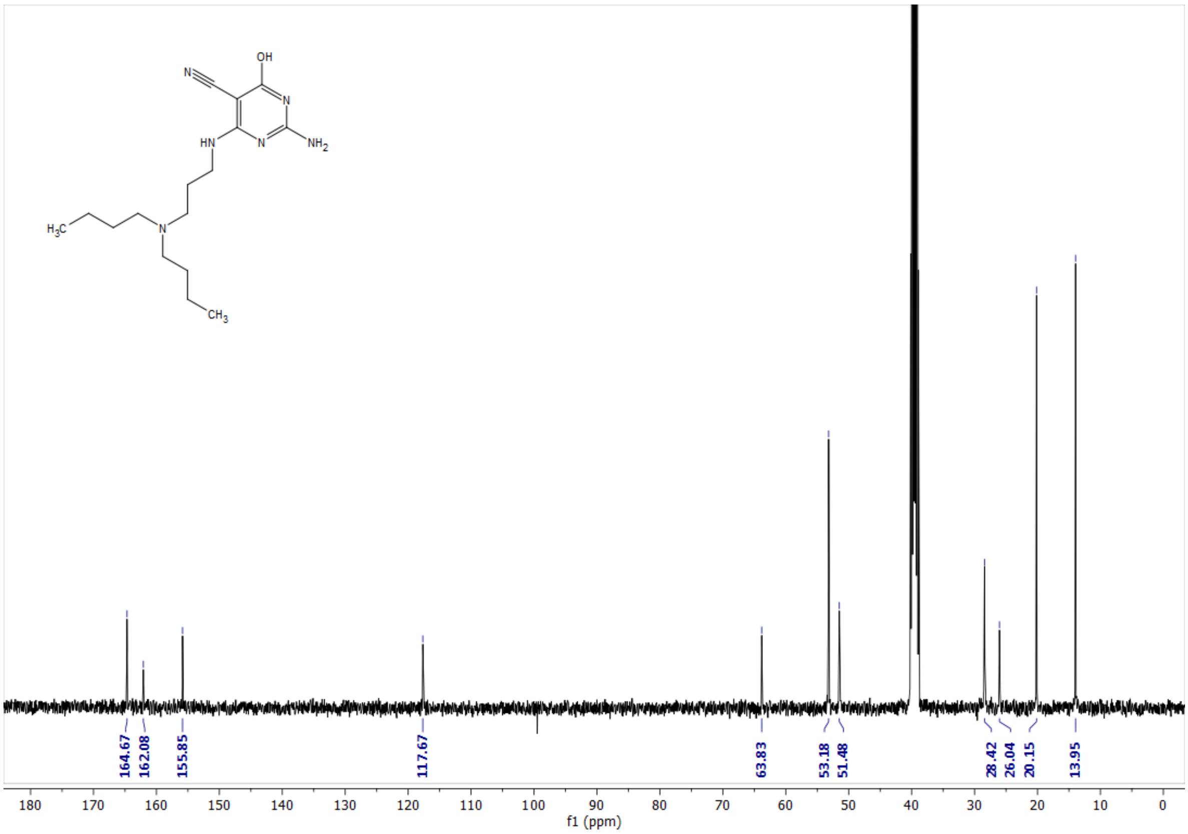

Figure S33. ^{13}C-NMR (101 MHz, DMSO-d$_6$) spectrum of compound **16e**

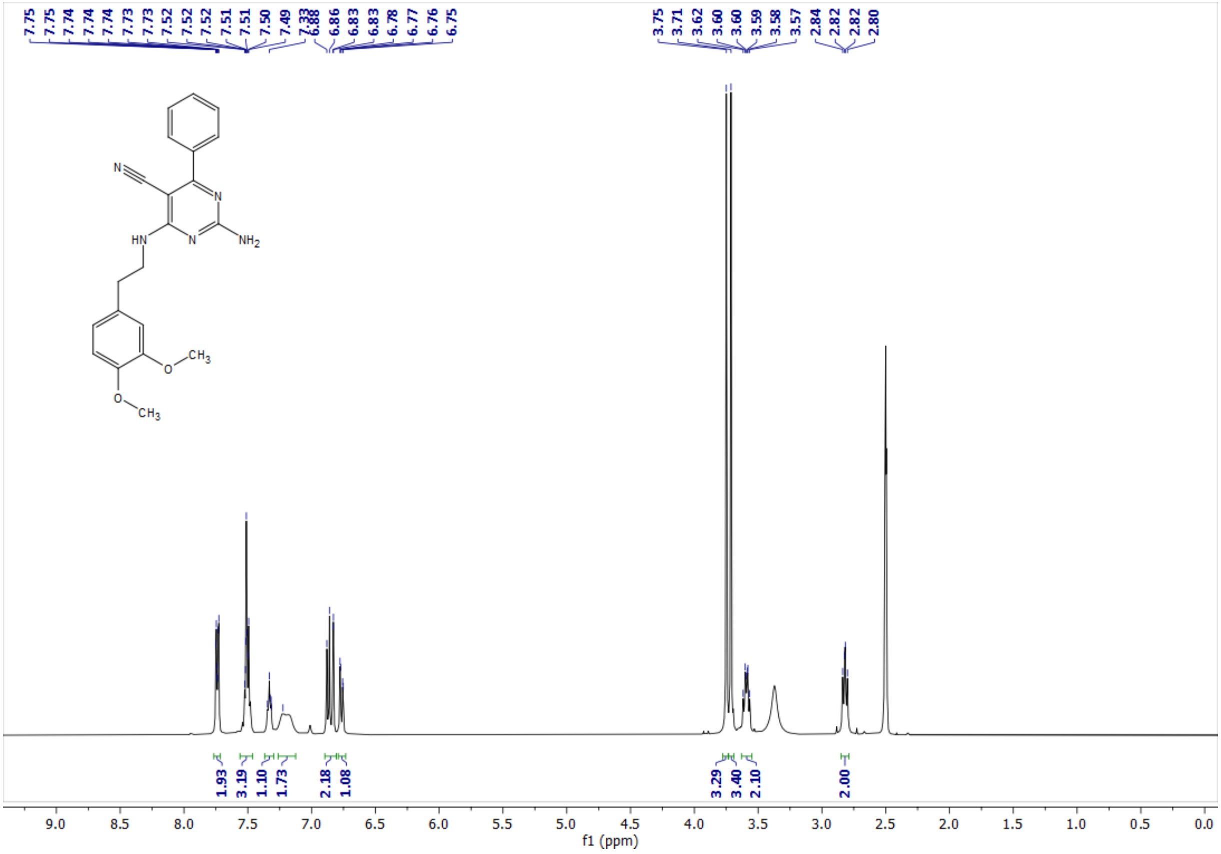

Figure S34. ^{1}H-NMR (400 MHz, DMSO-d$_6$) spectrum of compound **17a**

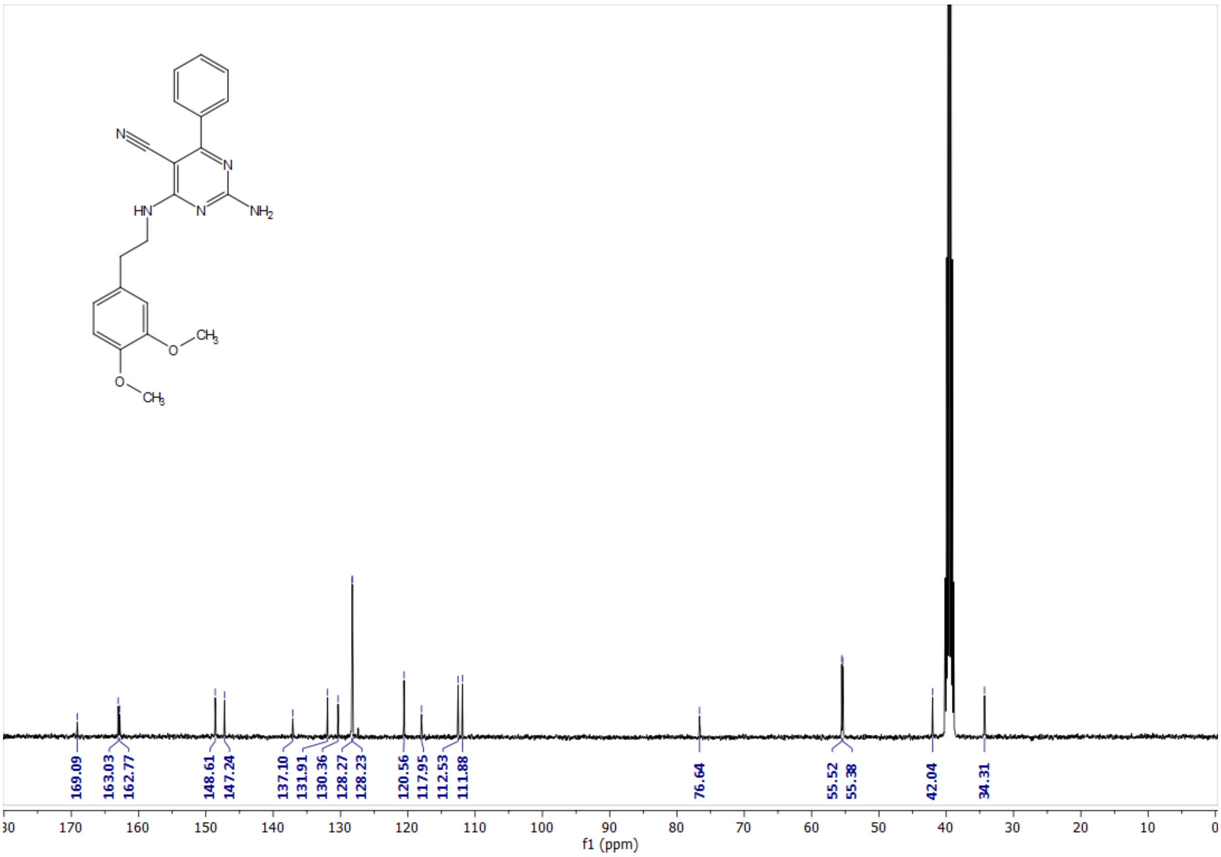

Figure S35. ^{13}C-NMR (101 MHz, DMSO-d$_6$) spectrum of compound **17a**

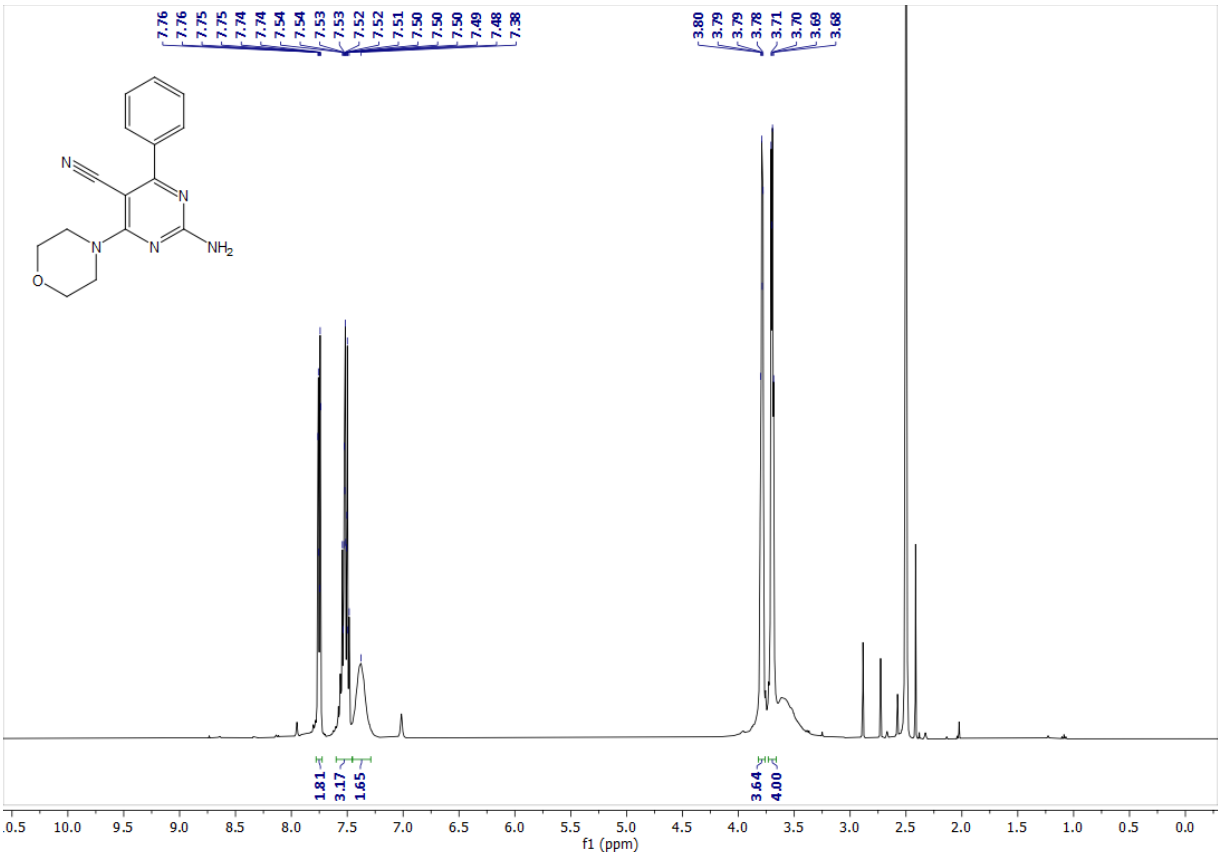

Figure S36. ^{1}H-NMR (400 MHz, DMSO-d$_6$) spectrum of compound **17b**

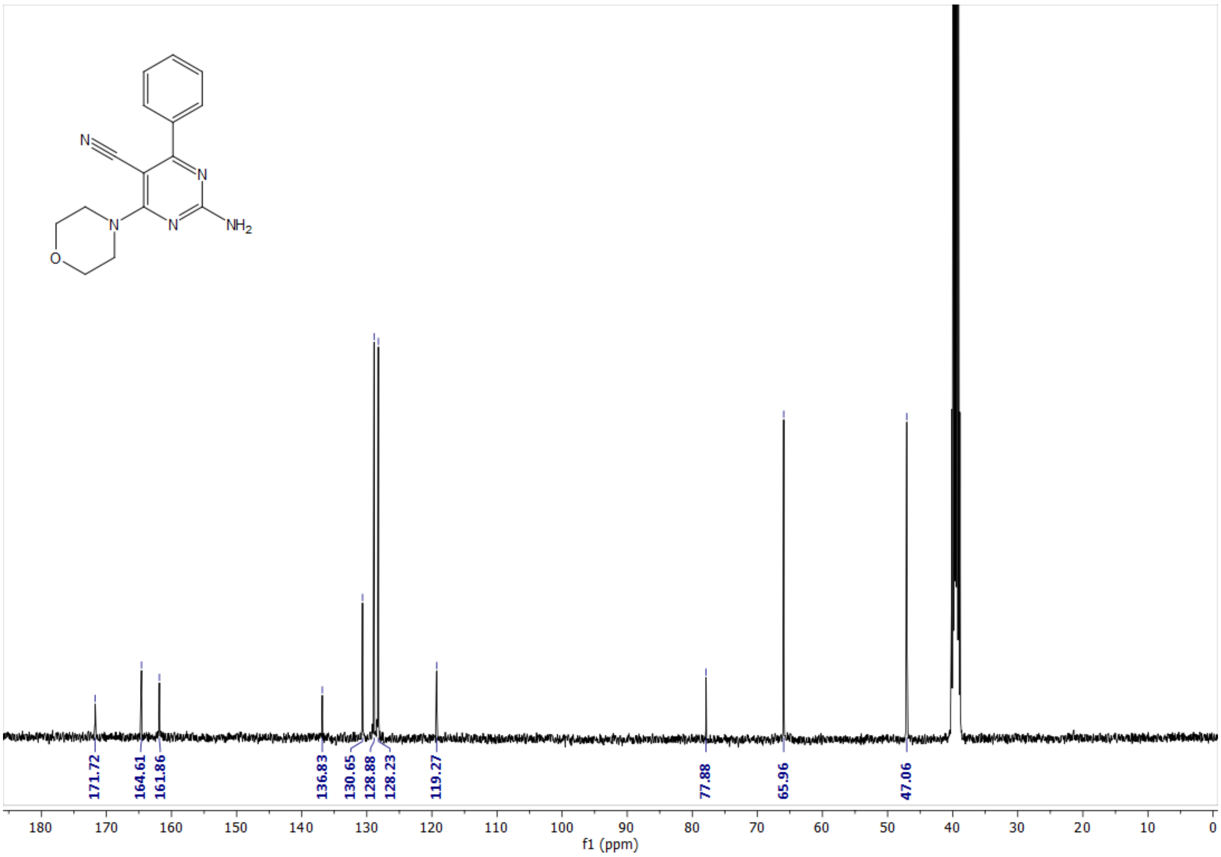

Figure S37. ^{13}C-NMR (101 MHz, DMSO-d$_6$) spectrum of compound **17b**

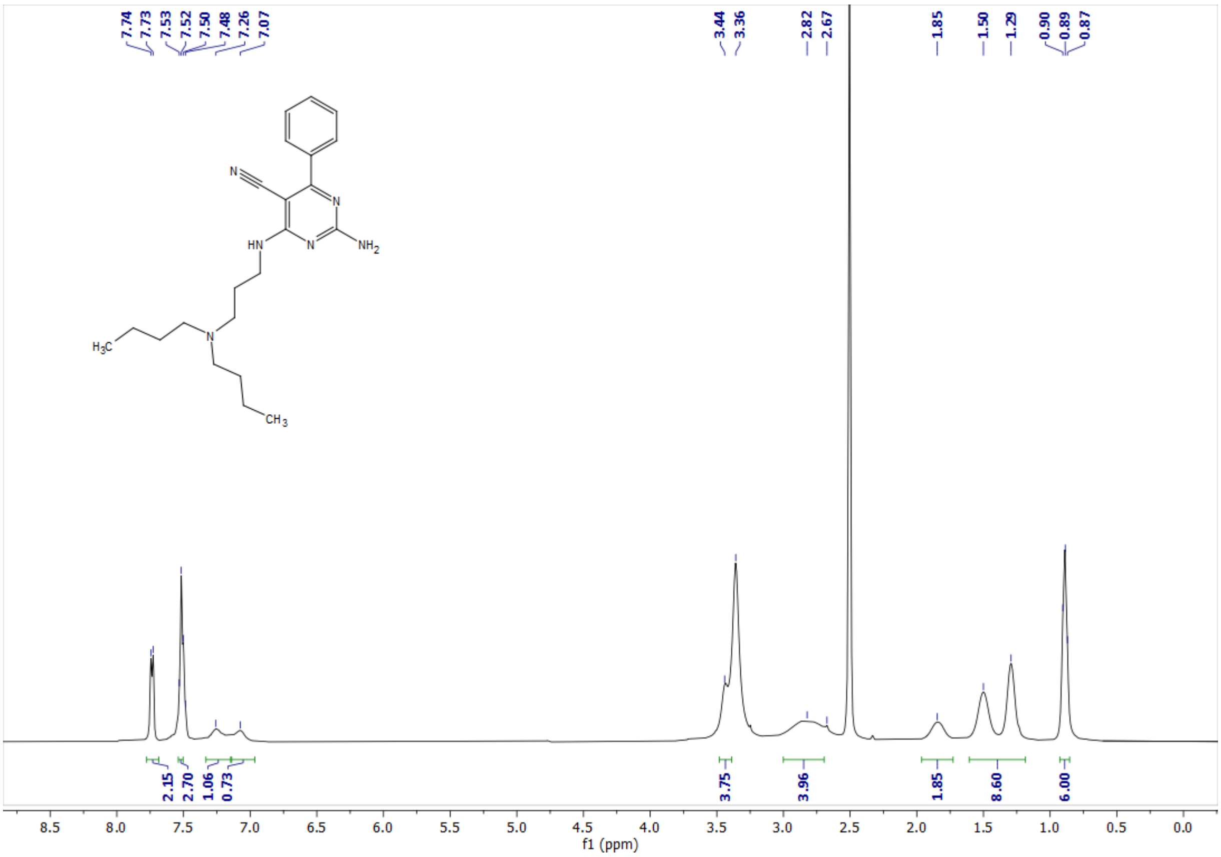

Figure S38. ^{1}H-NMR (400 MHz, DMSO-d$_6$) spectrum of compound **17e**

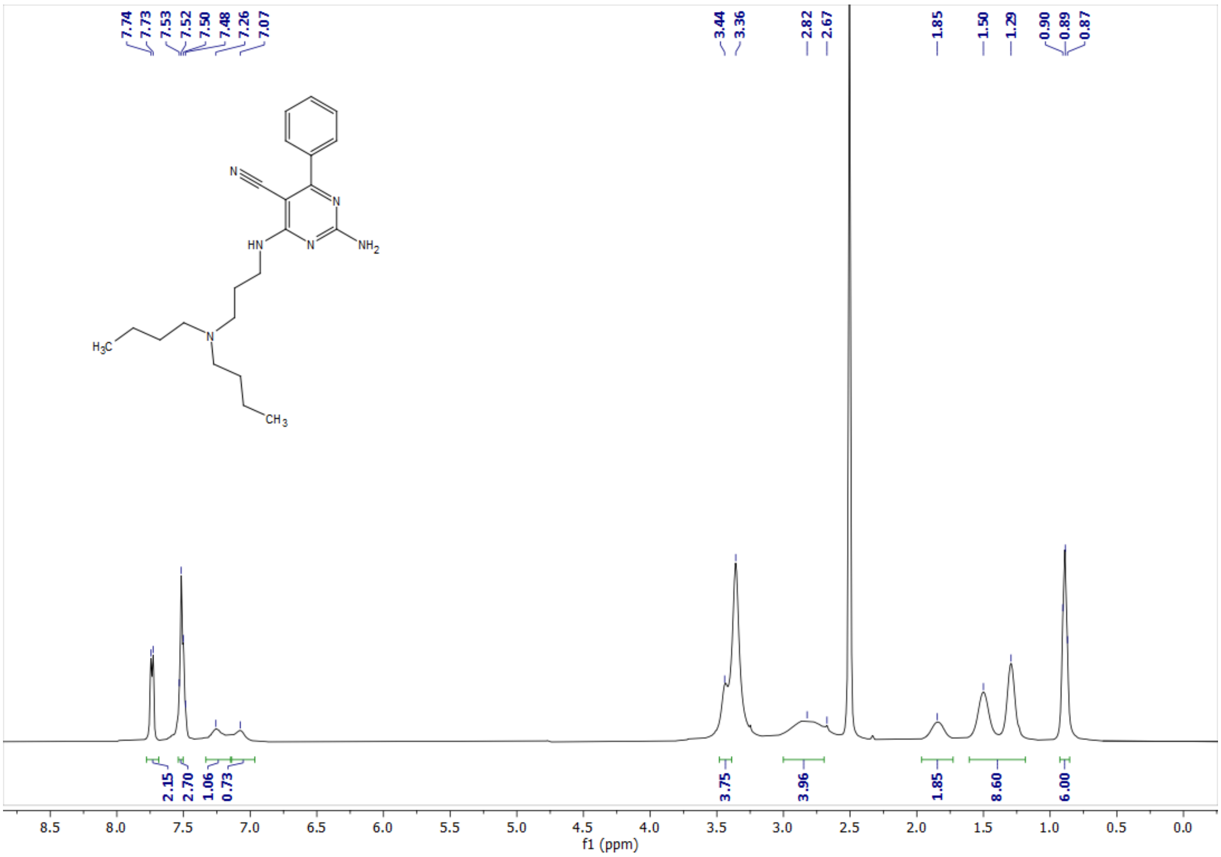

Figure S39. ^{13}C-NMR (101 MHz, DMSO-d$_6$) spectrum of compound **17e**

Table S1. Predicted Absorption and distribution properties of compounds **13-17**[a]

		Pgp						Inhibitor			
**ML**	**Cpd**	Inh	Sub	**HIA**[b]	**F_{50}**[c]	**PPB**[d]	**BBB**[e]	**OATP1B1**	**OATP1B3**	**BCRP**	**MRP1**
99	**13a**	Yes	Yes	No	No	98	No	Yes	Yes	No	Yes
97	**13b**	Yes	No	No	No	77	No	Yes	Yes	No	Yes
101	**13c**	Yes	No	Yes	No	99	No	Yes	Yes	No	Yes
91	**14a**	Yes	No	Yes	Yes	99	No	No	No	No	Yes
95	**14b**	No	No	Yes	Yes	93	No	No	No	No	Yes
90	**14c**	Yes	No	Yes	No	98	No	No	No	No	Yes
172	**14d**	No	No	Yes	No	88	No	No	Yes	No	Yes
170	**14e**	No	No	Yes	No	82	No	No	Yes	No	Yes
183	**14f**	No	No	Yes	No	65	No	Yes	Yes	No	Yes
70	**15a**	No	No	Yes	Yes	54	No	No	No	No	Yes
72	**15b**	No	No	Yes	Yes	46	No	No	No	No	Yes
73	**15c**	No	No	Yes	Yes	91	No	No	No	No	Yes
203	**16a**	No	No	Yes	Yes	93	No	No	No	No	Yes
209	**16b**	No	No	Yes	Yes	41	No	No	No	No	Yes
206	**16e**	No	No	No	No	37	No	No	Yes	No	Yes
210	**17a**	Yes	No	Yes	No	98	No	No	No	No	Yes
217	**17b**	No	No	Yes	Yes	82	No	No	No	No	Yes
214	**17e**	Yes	No	Yes	No	77	No	No	Yes	No	Yes

[a]The flags "Yes" and "No" have been assigned according to the calculated probability value (Yes: probability ≥ 70%; No: probability < 70%). [b]Human Intestinal Absorption ≥ 30%. [c]Bioavailability ≥ 50%. [d]Plasma protein binding percentage. Optimal value below 90%. [e]Blood-brain barrier penetration.

Table S2. Predicted metabolism and excretion properties of compounds **13-17.**[a]

		CYP inhibitor							CYP substrate							
**ML**	**Cpd**	1A2	2C19	2C9	2D6	3A4	2B6	2C8	1A2	2C19	2C9	2D6	3A4	2B6	**CL**[b]	**$T_{½}$**[c]
99	**13a**	No	Yes	Yes	No	Yes	Yes	Yes	Yes	Yes	Yes	No	Yes	No	6.2	0.8
97	**13b**	No	Yes	Yes	No	No	No	No	No	No	No	No	No	No	6.5	0.7
101	**13c**	No	Yes	Yes	No	No	No	Yes	No	No	No	No	Yes	Yes	6.7	0.6
91	**14a**	Yes	Yes	Yes	No	Yes	No	Yes	Yes	Yes	Yes	No	Yes	No	5.6	0.8
95	**14b**	Yes	Yes	Yes	No	No	No	Yes	No	No	Yes	No	No	No	4.9	1.0
90	**14c**	Yes	Yes	Yes	No	No	No	Yes	No	No	No	No	No	No	5.2	0.8
172	**14d**	No	No	No	Yes	No	Yes	No	Yes	No	No	Yes	Yes	No	5.5	0.6
170	**14e**	No	No	No	Yes	No	Yes	No	Yes	No	No	Yes	Yes	No	5.8	0.5
183	**14f**	No	No	No	Yes	No	Yes	No	Yes	No	No	Yes	Yes	No	5.2	0.5
70	**15a**	No	Yes	No	No	Yes	No	Yes	Yes	Yes	Yes	Yes	Yes	No	3.6	0.8
72	**15b**	No	No	No	No	No	No	No	No	No	Yes	No	No	No	3.1	0.8
73	**15c**	No	Yes	No	No	No	No	No	No	No	No	No	No	No	3.6	0.8
203	**16a**	No	Yes	Yes	No	Yes	No	Yes	Yes	Yes	Yes	No	Yes	No	5.3	1.1
209	**16b**	No	No	No	No	No	No	No	No	No	No	No	No	No	5.1	1.0
206	**16e**	No	No	No	Yes	No	No	No	Yes	No	No	Yes	Yes	No	5.9	0.8
210	**17a**	Yes	Yes	Yes	No	Yes	Yes	Yes	Yes	Yes	No	No	Yes	No	5.8	1.1
217	**17b**	No	No	No	No	No	No	No	No	No	No	No	No	No	5.4	0.9
214	**17e**	No	No	No	Yes	No	Yes	No	Yes	No	No	Yes	Yes	No	6.1	0.5

[a] see Table S1. [b]Plasma clearance. Values are expressed in ml/min/Kg. [c]Half life. Values are expressed in hours.

Table S3. Predicted toxicity profile of compounds **13-17.**[a]

**Cpd**	**13a**	**13b**	**13c**	**14a**	**14b**	**14c**	**14d**	**14e**	**14f**	**15a**	**15b**	**15c**	**16a**	**16b**	**16e**	**17a**	**17b**	**17e**
hERG blocker	No	No	No	No	No	**Yes**	Yes	Yes	Yes	Yes	No	Yes	No	No	Yes	No	No	Yes
DILI[b]	Yes	Yes	No	Yes	Yes	No	Yes	No	No	Yes	Yes	Yes	Yes	Yes	No	Yes	Yes	No
Mutagenic	No	No	No	No	No	No	No	No	No	No	No	No	No	No	No	No	No	No
ROA[c]	No	No	No	No	No	No	No	No	No	No	No	No	No	No	No	No	No	No
Skin sensitizer	No	No	No	No	No	No	Yes	Yes	Yes	No	Yes	No	No	No	Yes	No	No	Yes
Carcinogenic	No	Yes	No	No	No	No	No	No	No	No	No	No	No	Yes	No	No	No	No
Eye corrosive	No	No	No	No	No	No	No	Yes	No	No	No	No	No	No	No	No	No	No
Eye irritant	Yes	Yes	No	Yes	Yes	No	No	Yes	No	No	Yes	No	Yes	Yes	Yes	No	Yes	No
Respiratory	Yes	Yes	Yes	Yes	Yes	Yes	Yes	Yes	Yes	No	No	No	Yes	No	Yes	No	No	Yes
Hepatotoxic	No	Yes	Yes	No	No	Yes	No	No	Yes	Yes	Yes	Yes	No	Yes	No	Yes	Yes	Yes
Nephrotoxic	No	Yes	Yes	No	Yes	Yes	Yes	Yes	Yes	Yes	No	Yes	No	No	Yes	No	No	Yes
Ototoxic	No	No	No	No	No	No	No	No	Yes	No	No	No	No	No	No	No	No	No
Hematotoxic	No	No	No	No	No	No	No	No	No	No	No	No	No	No	No	No	No	No
Genotoxic	Yes	No	Yes	Yes	No	Yes	No	No	No	Yes	Yes	Yes	No	Yes	No	Yes	Yes	No
RPMI-8226	No	No	No	No	No	No	No	No	No	No	No	No	No	No	No	No	No	No
A549	No	No	No	No	No	No	No	No	No	No	No	No	No	No	No	No	No	No
HEK293	No	No	No	No	No	No	No	Yes	Yes	No	No	No	No	No	Yes	No	No	Yes
Neurotoxic	No	No	No	Yes	Yes	Yes	Yes	Yes	Yes	No	No	No	No	Yes	Yes	Yes	Yes	Yes

[a]see Table S1. [b]Drug induced liver injury. [c]Rat oral acute toxicity.

Table S4. Predicted Toxicity pathways of compounds **13-17.**[a]

	**13a**	**13b**	**13c**	**14a**	**14b**	**14c**	**14d**	**14e**	**14f**	**15a**	**15b**	**15c**	**16a**	**16b**	**16e**	**17a**	**17b**	**17e**
**NR-AhR**	No	No	No	Yes	No	No	No	No	Yes	No	No	No	No	No	Yes	Yes	No	Yes
**NR-AR**	No	No	No	No	No	No	No	No	No	No	No	No	No	No	No	No	No	No
**NR-AR-LBD**	No	No	No	No	No	No	No	No	No	No	No	No	No	No	No	No	No	No
**NR-Aromatase**	No	No	No	No	Yes	No	No	No	No	No	No	No	No	No	No	No	No	No
**NR-ER**	No	No	No	No	No	No	No	No	No	No	No	No	No	No	No	No	No	No
**NR-ER-LBD**	No	No	No	No	No	No	No	No	No	No	No	No	No	No	No	No	No	No
**NR-PPARγ**	No	No	No	No	No	No	No	No	No	No	No	No	No	No	No	No	No	No
**SR-ARE**	No	No	No	No	No	No	No	No	No	No	No	No	No	No	No	No	No	No
**SR-ATAD5**	No	No	No	No	No	No	No	No	No	No	No	No	No	No	No	No	No	No
**SR-HSE**	No	No	No	No	No	No	No	No	No	No	No	No	No	No	No	No	No	No
**SR-MMP**	No	No	Yes	No	No	Yes	No	No	No	No	No	Yes	No	No	No	Yes	Yes	No
**SR-p53**	No	No	No	No	No	No	No	No	No	No	No	No	No	No	No	No	No	No

[a]see Table S1. [b]Drug induced liver injury.
